# Harnessing stemness and PD-L1 expression by AT-rich interaction domain-containing protein 3B in colorectal cancer

**DOI:** 10.7150/thno.44147

**Published:** 2020-05-15

**Authors:** Tsai-Tsen Liao, Chun-Chi Lin, Jeng-Kae Jiang, Shung-Haur Yang, Hao-Wei Teng, Muh-Hwa Yang

**Affiliations:** 1 Graduate Institute of Medical Sciences, College of Medicine, Taipei Medical University, Taipei, Taiwan; 2 Cell Physiology and Molecular Image Research Center, Wan Fang Hospital, Taipei Medical University; 3 Institute of Clinical Medicine, National Yang-Ming University, Taipei, Taiwan; 4 School of Medicine, National Yang-Ming University, Taipei, Taiwan; 5 Division of Colorectal Surgery, Department of Surgery, Taipei Veterans General Hospital, Taipei, Taiwan; 6 Department of Surgery, National Yang-Ming University Hospital, I-Lan, Taiwan; 7 Division of Medical Oncology, Department of Oncology, Taipei Veterans General Hospital, Taipei, Taiwan; 8 Cancer Progression Research Center, National Yang-Ming University, Taipei, Taiwan

**Keywords:** ARID3B, cancer stem cell, colorectal cancer, Notch, programmed death ligand 1

## Abstract

**Background and Aims**: Cancer stem cells (CSCs) have been shown to be responsible for the tumor initiation, metastasis, and therapeutic resistance of colorectal cancer (CRC). Recent studies have also indicated the importance of CSCs in escaping immune surveillance. However, the coordinated epigenetic control of the stem cell signature and the key molecule(s) involved in immunosurveillance of colorectal CSCs (CRCSCs) are unclear. Here, we investigated the role of a histone modifier, AT-rich interaction domain-containing protein 3B (ARID3B), in CRC.

**Methods**: CRC patient-derived xenografts (PDXs) with knockout of ARID3B induced by CRISPR/Cas9 *in vivo* were used*.* Molecular/cellular biology assays were performed. Clinical data obtained from The Cancer Genome Atlas, as well as from our cohort (Taipei Veterans General Hospital), were analyzed.

**Results**: ARID3B was crucial for the growth of CRC, and ARID3B promoted the stem-like features of CRC. Mechanistically, ARID3B activated Notch target genes, intestinal stem cell (ISC) genes, and programmed death-ligand 1 (PD-L1) through the recruitment of lysine-specific demethylase 4C (KDM4C) to modulate the chromatin configuration for transcriptional activation. Clinical sample analyses showed that the coexpression of ARID3B and the Notch target HES1 correlated with a worse outcome and that ARID3B and PD-L1 were highly expressed in the consensus molecular subtype 4 of CRC. Pharmacological inhibition of KDM4 activity reversed the ARID3B-induced signature.

**Conclusion**: We reveal a noncanonical Notch pathway for activating Notch target genes, ISC genes, and PD-L1 in CRC. This finding explains the immune escape of CRCSCs and indicates a potential group that may benefit from immune checkpoint inhibitors. Epigenetic drugs for reversing stem-like features of CRC should also be investigated.

## Introduction

Colorectal cancer (CRC) is one of the most common and deadliest diseases worldwide 1. The carcinogenic process of CRC occurs due to the accumulation of genetic and epigenetic changes that transform colon epithelial cells into heterogeneous adenoma-carcinoma cells 2. Improvements in diagnosis, screening, and treatment have resulted in increased long-term survival rates for patients with early CRC. However, the prognosis of CRC patients with advanced disease remains poor, and effective therapies for eliminating latent disseminated/refractory CRC are still unsatisfactory 3-5. Recently, the interplay between cancer cells and host immune cells has been extremely attractive for cancer researchers owing to the success of immune checkpoint inhibitors (ICIs) in advanced cancers. Different ICIs, including programmed cell death protein-1 (PD-1)-blocking monoclonal antibodies (mAbs) and programmed death-ligand 1 (PD-L1)-targeted mAbs, have been approved to treat multiple types of cancers, including melanoma, lung cancer, and head and neck cancer 6-8. Unfortunately, in metastatic CRC, anti-PD1/PD-L1 antibodies benefit only a limited subset of patients with deficient mismatch repair that causes a high level of microsatellite instability 9. Finding an optimal patient population for extending the application and improving the efficacy of ICIs is an urgent but unmet need for advanced CRC.

Acquisition of the stem-like properties in CRC is critical for metastasis and therapeutic resistance 10-12. Colorectal cancer stem cells (CRCSCs) exhibit characteristics that are similar to intestinal epithelial stem cells (ISCs), although CRCSCs may not necessarily be derived directly from normal ISCs 13. CRCSCs and ISCs share several important signaling mechanisms, such as the Wnt 14 and Notch pathways 15, and express similar markers, such as Lgr5 16 and CD44 17. However, elimination of CRCSCs is therefore difficult because of the similarities between ISCs and CRCSCs. Moreover, CSCs have been indicated to escape immune surveillance, and immune surveillance thereby enriches the CSC subpopulation in tumors 18, 19. Recent studies have shown that PD-L1 is particularly highly expressed in CSCs 20, 21. The expression of PD-L1 on CSCs not only promotes their stem-like properties but also contributes to immune evasion 22-24. Although the mechanisms of oncogenic signaling activation-mediated PD-L1 expression are gradually being elucidated 19, 24, 25, our understanding of the epigenetic regulation contributing to PD-L1 expression in CRCSCs remains limited. Furthermore, the low percentage of PD-L1 staining in whole tumors may not exclude the existence of PD-L1-expressing CSCs. Elucidation of the mechanisms underlying acquisition of stem-like properties and PD-L1 expression in the CSCs of CRC will help identify optimal populations and develop strategies to improve the efficacy of ICIs for eradicating CRCSCs. The AT-rich interaction domain (ARID) family proteins contain three members (ARID3A, ARID3B, ARID3C) that harbor a distinctive DNA-binding domain named ARID. Their encoded proteins have similar amino acid sequences characterized by an extended ARID (with additional alpha-helices at the N- and C-termini of the core ARID) 26-27. ARID3 members have been implicated in the regulation of the cell cycle, gene expression, embryonic development, differentiation, chromatin remodeling, and transcriptional regulation 26,28-30. ARID3B is essential during embryonic development, and deletion of Arid3b in mice leads to multiple abnormalities and embryonic lethality 31, 32. In human cancers, ARID3B is considered an oncoprotein, and overexpression of ARID3B has been noted in ovarian cancer 33, neuroblastoma 34, and breast cancer 35. In our recent study, we linked ARID3B to histone methylation dynamics and elucidated the mechanisms underlying ARID3B-regulated stemness factors in head and neck cancer cells 36. Here, we showed that in CRC, the histone modifier ARID3B harnesses the expression of target genes, including the ISC genes, Notch target genes, and PD-L1, which highlights a mechanism for generating PD-L1-expressing CRCSCs. These results provide insights into the potential strategies for advanced CRC, such as administering histone demethylase inhibitors to suppress the activation of ARID3B-mediated target genes or administering ICIs to eliminate PD-L1-expressing CRCSCs.

## Materials and Methods

**Cell lines and plasmids.** Four human CRC cell lines (HCT-15, HT-29, CaCo2, and SW480) and HEK-293T cells were used in this study. The cell lines were authenticated before the experiments were performed. The pCDH-ARID3B plasmid was previously described 36. The CRISPR-Cas9 plasmids were purchased from Addgene (Cambridge, MA). The construction of the CRISPR-Cas9 plasmid for knocking out ARID3B and other plasmid information is detailed in Table S1. The reporter plasmids 4x wt CBF1 Luc and 4x mt CBF1 Luc, which contain four repeats of the wild-type CBF1 binding site, were provided by Dr. Tien-Shun Yeh (National Yang-Ming University).

**Soft agar colony formation assay.** The wells of a six-well dish were coated with 1 ml of a bottom agar mixture (DMEM containing 15% FBS, 0.5% agar, and 1% penicillin-streptomycin). After the bottom layer solidified, 1 ml of a top agar-medium mixture (DMEM containing 15% FBS, 0.3% agar, and 1% penicillin-streptomycin) containing 5,000 cells was added, and the dishes were incubated at 37°C for 2 weeks. The plates were stained with crystal violet, and the number of colonies was counted in a 10x low power field (LPF). Ten LPFs per well and three wells for each experimental condition were counted. The experiment was conducted with three independent biological replicates, and each biological replicate contained three technical replicates.

**Cell migration and invasion assays.** Cell migration and invasion were evaluated using a transwell with an 8-μm filter membrane-containing upper chamber (Greiner Bio-One, Inc., Monroe, NC). Cells (1 x 10^5^ for HT-29 and 2 x 10^5^ for HCT-15) suspended in 100 μl of culture medium containing 0.5% FBS were applied to the upper chamber, and 600 μl of medium containing 15% FBS was added to the lower chamber. For the migration assay, the uncoated upper chambers were used for experiments. For the invasion assay, the upper chambers were covered with Matrigel (Collaborative Research, Inc., Boston, MA) before seeding the cells. After 24 h, the cells on the upper side of the filter were removed, and the cells that remained adherent to the underside of the membrane were fixed in 4% formaldehyde and stained with Hoechst 33342 dye. The number of migrated cells was counted using a fluorescence microscope. The experiment was conducted with three independent biological replicates, and each biological replicate contained three technical replicates.

**cDNA microarrays.** HCT15-ARID3B-Cas9 and HCT15-Ctrl cells were analyzed using Human Genome U133 Plus 2.0 chips (Affymetrix). (Thermo Fisher Scientific, Inc., Waltham, MA). Biotinylated cRNA was prepared according to the standard Affymetrix protocol, and hybridization was performed according to the standard Affymetrix protocol and scanned with the Affymetrix GeneChip® Scanner 3000 7G. The data were extracted using Microarray Analysis Suite 5.0. Gene Ontology analysis was performed with DAVID bioinformatics.

**Quantitative RT-PCR.** Quantitative PCR was performed using the StepOne-Plus real-time PCR system (Applied Biosystems, Inc., Foster City, CA). The primer sequences used for real-time PCR experiments are listed in Table S2. The experiment was conducted with three independent biological replicates, and each biological replicate contained two technical replicates.

**Electrophoretic mobility shift assay (EMSA).** Oligonucleotides containing 3x conserved CBF1 binding sequences (GTGGGAA) were labeled with biotin and incubated with nuclear extracts harvested from HT29-ARID3B or HT29-Vec cells. The mixture was electrophoresed and transferred onto a nylon membrane, and the signals were detected by streptavidin-HRP. For the supershift assay, 2 μg of the anti-ARID3B antibody was added to the reaction mixture and electrophoresed. For the competition assay, excess amounts of unlabeled competitors were added before the labeled probes. For the pull-down assay, the protein-probe binding mixture was pulled down by streptavidin-agarose beads, and the protein was further analyzed by Western blotting.

***In vitro* histone demethylase activity assay.** For analysis of histone demethylase activity *in vitro*, 238 ng of recombinant KDM4C (1-460 aa, ab167940; Abcam) was incubated with 2 μg of biotin-labeled H3K9me3 peptides at 37°C for 3 h in histone demethylation buffer (50 mM HEPES-KOH (pH 8.0), 50 μM Fe(SO_4_)_2_, 1 mM a-ketoglutarate, and 2 mM ascorbate). Substrate methylation levels were analyzed by dot blot with specific antibodies. The antibodies used in the experiment are listed in Table S3.

**Chromatin immunoprecipitation (ChIP) and sequential ChIP.** Immunoprecipitation (IP) was carried out using a Pierce magnetic ChIP kit (Thermo Scientific, Rockford, IL) according to the manufacturer's instructions. Briefly, the cells were crosslinked with 1% formaldehyde and harvested. The nuclear fractions were resuspended and subjected to sonication, and the lysates were then incubated with magnetic beads conjugated to specific antibodies against different proteins or IgG control. The DNA-protein complexes were then eluted, and specific regions were amplified by PCR. For sequential ChIP experiments, the supernatant was collected, diluted 1:500 in IP buffer, and subjected to the IP procedure again, and the regions of interest were amplified. The experiment was conducted with three independent biological replicates, and each biological replicate contained two technical replicates. One representative experiment of three independent experiments is shown in the main figures, and the other two experiments are shown in the supplementary figures. The antibodies used in the experiment are listed in Table S3, and the primers used in the experiments are listed in Table S4.

**Construction of tissue microarrays of colorectal cancer samples and immunohistochemistry.** This study was approved by the Institutional Review Board of the Taipei Veterans General Hospital. We enrolled two sets of tissue microarrays, one composed of samples from 130 CRC patients and the other containing 15 pairs of primary tumors with liver metastasis. The pathological staging was performed according to the 6^th^ edition of the American Joint Committee on Cancer system. For immunohistochemistry (IHC), sections were deparaffinized in xylene and rehydrated in a descending ethanol series. The primary antibodies used in the study are listed in Table S3. Bound antibodies were visualized using the Novolink polymer detection system (Leica Biosystems Newcastle, Ltd.), and diaminobenzidine was used as a chromogen. Immunopositivity was evaluated by two experts blinded to the clinical information. A semiquantitative analysis of the stained sections was performed according to the immunoreactive score (IRS) 37. For ARID3B, a score of 0-8 was categorized as low expression, whereas 9-12 was categorized as high expression; for KDM4C and HES1, a score of 0-3 was considered to indicate low expression, and 4-12 was categorized as high expression. For analysis of the PD-L1 expression, the intensity of PD-L1 expression was scored as 0 (absent), 1 (weak), 2 (moderate), or 3 (strong) 38.

***In vivo* gene targeting and tumorigenicity assay.** The animal studies were approved by the Committee on the Ethics of Animal Experiments at Taipei Veterans General Hospital (approval IACUC No. 2018‐191). The established process of PDXs was performed as described previously 39. Briefly, the residual CRC specimens were first rinsed twice and immersed in Matrigel (Becton‐Dickinson) at 37°C. The tumors were cut into 1 mm^3^ pieces and subcutaneously implanted in 4‐week‐old female nude mice to establish PDXs. *In vivo* gene silencing was performed using the IDLV‐CRISPR/Cas9 system 40. PDXs at less than five passages were intratumorally injected with 1.8 × 10^8^ virus particles one‐week after tumor implantation. For virus production, 15 μg targeting vector, 10 μg pBK43 integrase‐deficient packaging cassette, 5 μg pMD2.G envelope plasmid (#12259, Addgene) and 2.5 μg pRSV‐Rev plasmid (#12253, Addgene) were introduced into 293T cells by transfection. For evaluation of the tumorigenicity of the CRC cell lines, a xenograft assay was performed by inoculating 1 × 10^5^ or 1 × 10^6^ cells into the subcutaneous region of nude mice.

**CMS classification.** The data set provided by the Colorectal Cancer Subtyping Consortium that corresponded to GSE37892 and PETACC3 were downloaded from the Synapse data portal. The PETACC3 dataset (ArrayExpress E‐MTAB‐990), generated by the Almac Affymetrix custom chip, did not contain the *CD274* gene probe. Therefore, *CD274* could not be analyzed in this dataset.

**Statistical analysis.** The numerical results are presented as the mean ± S.D. A two-tailed independent Student's *t*-test was used to compare the continuous variables between the two groups by Prism 5 software. Pearson's chi-squared test was used to evaluate the dichotomous variables between the two groups by IBM Statistical Product and Service Solutions (SPSS) version 22. A Kaplan-Meier estimation and the log-rank test were used to compare survival between the patient groups by SPSS version 22. All statistical data were derived from three independent biological replicates, and each experiment contained two technical replicates. The level of statistical significance was set to p ≤ 0.05 for all tests.

**Data availability.** All relevant data are available from the corresponding author upon reasonable request. The datasets obtained from the cDNA microarray of HCT cells subjected to CRISPR/Cas9-mediated ARID3B depletion (HCT15-ARID3B-Cas9) and control cells were deposited in the Gene Expression Omnibus (GEO) database under accession number GSE92838 (secure token: ulwzoaeabjobzcr, https://www.ncbi.nlm.nih.gov/geo/query/acc.cgi?acc=GSE92838). For CMS classification, the data set provided by the Colorectal Cancer Subtyping Consortium that corresponded to GSE37892 and PETACC3 were downloaded from the Synapse data portal. The PETACC3 dataset (ArrayExpress E‐MTAB‐990) was generated by the Almac Affymetrix custom chip, which did not contain the *CD274* gene probe. Therefore, *CD274* could not be analyzed in this dataset. The other public databases used in GSEA are listed as follows: the gene expression profile in colon cancer patient samples with different clinical statuses (GSE17538) 41; the gene expression profile of CD133+ and CD133- samples isolated from colon cancer patients (GSE34053); and the GSI-NOTCH gene set containing the genes downregulated by treatment with a gamma secretase inhibitor 42.

## Results

**ARID3B is critical for the growth and progression of colorectal cancer.** Compared to the extensive studies of genetic aberrations during CRC tumorigenesis and progression, few analyses of the epigenetic regulation of CRC have been performed. Increasing evidence supports the role of the histone modifier ARID3B in the tumorigenesis of different types of cancers, including ovarian cancer, neuroblastoma, and head and neck cancer, by regulating stemness-related genes 33, 34, 36. Because the stemness signatures and their regulatory mechanisms are distinct among different cancers 43, 44, we investigated the role of ARID3B in the tumorigenesis and stemness of CRC. To examine whether ARID3B is crucial for CRC growth, we established three patient-derived xenografts (PDXs) from CRC patients. The characteristics of these three patients for generating PDXs are listed in Table S5. The PDXs for the experiments were all at less than 5 passages. We used immunohistochemistry to examine the expression of ARID3B in the three patient samples to generate the PDXs (Figure S1A). The results showed that all three samples expressed a high level of ARID3B, which indicates the importance of ARID3B in tumor initiation and propagation and justifies the application of CRISPR/Cas 9 to deplete ARID3B in these tumors for subsequent experiments.

**Fig 1:**
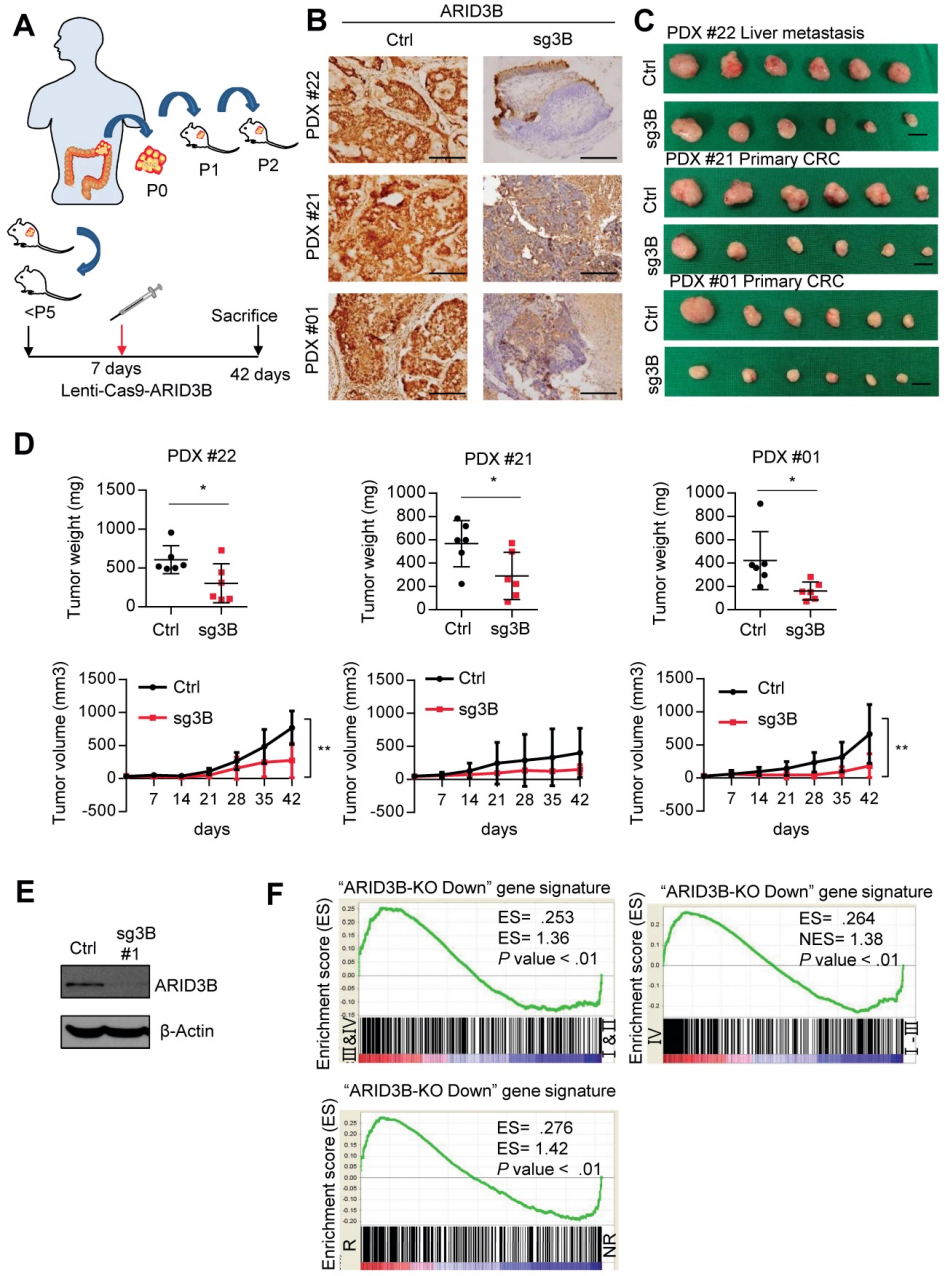
ARID3B is critical for CRC tumor growth and progression. **A** Schematic of PDX generation and *in vivo* knockout of ARID3B. **B** Representative images showing the diminishment of ARID3B in PDXs that received the ARID3B knockout vector (sg3B) compared to the control vector (Ctrl) *in vivo*. Scale=200 µm.** C** Representative photos of PDXs that received sg3B or Ctrl. n=6 for each group. Scale bar=1 cm. **D** The tumor weight and tumor size were both decreased with the PDXs receiving sg3B compared to Ctrl. n=6 for each group. **E** Western blot of ARID3B knockout in HCT-15 cells receiving CRISPR/Cas9 for depleting ARID3B vs. the control. β-actin was used as a loading control. **F** GSEA shows the positive correlation between the ARID3B-regulated signature and severe tumor staging (I & II vs. III & IV; IV vs. I - III) or CRC samples with recurrence (R) versus nonrecurrence (NR). ARID3B-KO Down, the genes downregulated ≥ 2.6-fold in ARID3B knockout cells. ES, enrichment score. NES, normalized enrichment score. For the panels in Fig. 1, data represent the mean ± S.E.M. ^*^p < 0.05, ^**^p < 0.01, ^***^p < 0.001. See also Figure S1 and Table S5.

We next depleted ARID3B in CRC PDXs by intratumoral injection of the integrase-deficient lentiviral vector (IDLV)-CRISPR/Cas9 system 40 into PDXs on the 7^th^ day after tumor inoculation. The mice were sacrificed on the 42^nd^ day, and the tumor samples were harvested for analyses. The schema of the PDX experiments is illustrated in Figure 1A. Immunohistochemical staining (IHC) confirmed the successful repression of ARID3B by IDLV-CRISPR/Cas9 injection in the xenografted tumors (Figure 1B). The analysis of xenograft tumor growth showed that suppression of ARID3B significantly inhibited tumor growth (Figure 1C-D). IHC staining of cleaved caspase-3 in the PDX tumors showed no significant difference between the ARID3B-depleted and control groups (Figure S1B), indicating the critical role of ARID3B in maintaining CRC growth without directly affecting the viability of tumor cells.

Because ARID3B is known as a chromatin modifier that simultaneously regulates the expression of multiple genes, we investigated the clinical impact of the ARID3B-regulated gene signature in CRC. We first defined the ARID3B-regulated signature in CRC cells. We examined the endogenous level of ARID3B in different CRC cell lines to select appropriate cells to deplete ARID3B. Of the cell lines analyzed, HT-29 exhibited the lowest level of endogenous ARID3B, whereas HCT-15 had the highest expression of ARID3B (Figure S1C). Therefore, we used CRISPR-Cas9 to target the CDS domain of ARID3B to deplete ARID3B in HCT-15 cells (Figure 1E and Figure S1D). We examined the expression of the ARID3 family proteins to validate the specificity of the sequences for ARID3B knockout used in this study. We also examined caspase-3/cleaved caspase-3 to evaluate the cellular toxicity of the knockout sequences. The results showed that the two sequences primarily targeted ARID3B without prominent cytotoxicity (Figure S1E). Next, HCT-15 cells with the Cas9-ARID3B #1 clone and HCT-15 control cells were analyzed by a cDNA microarray to define the ARID3B-regulated gene signature, which contained 472 genes that were downregulated ≥ 2.6-fold in the ARID3B-depleted cells compared with the control cells (Table S6). Gene set enrichment analysis (GSEA) was performed to investigate the association between the ARID3B-regulated signatures and CRC patient gene expression profiles from the public dataset (GSE17538), which contains the gene expression profile of tumor samples from 238 colorectal cancer patients 41. GSEA revealed that the ARID3B-regulated signature was significantly associated with an advanced stage (either AJCC stage III & IV vs. I & II or stage IV vs. stage I-III) and recurrence of CRC (Figure 1F). Altogether, the above results support the role of ARID3B in CRC growth, and the ARID3B-regulated gene signature correlates with CRC progression.

**ARID3B promotes the stem-like properties and intestinal stem cell signature of CRC cells.** We next explored whether ARID3B can promote the stem-like properties of CRC. Ectopic expression of ARID3B in HT-29 cells, which have low endogenous levels of ARID3B, promoted migration, increased colony formation, enhanced the sphere-forming ability, and enriched the CD44-positive population, whereas depletion of ARID3B in HCT-15 cells suppressed migration, attenuated colony formation and the sphere-forming ability, and reduced the CD44-positive population (Figure S2A-E). A xenotransplantation assay showed that the ectopic expression of ARID3B promoted tumor growth in HT-29 cells more prominently when inoculating a lower cell dose (Figure S2F).

**Fig 2:**
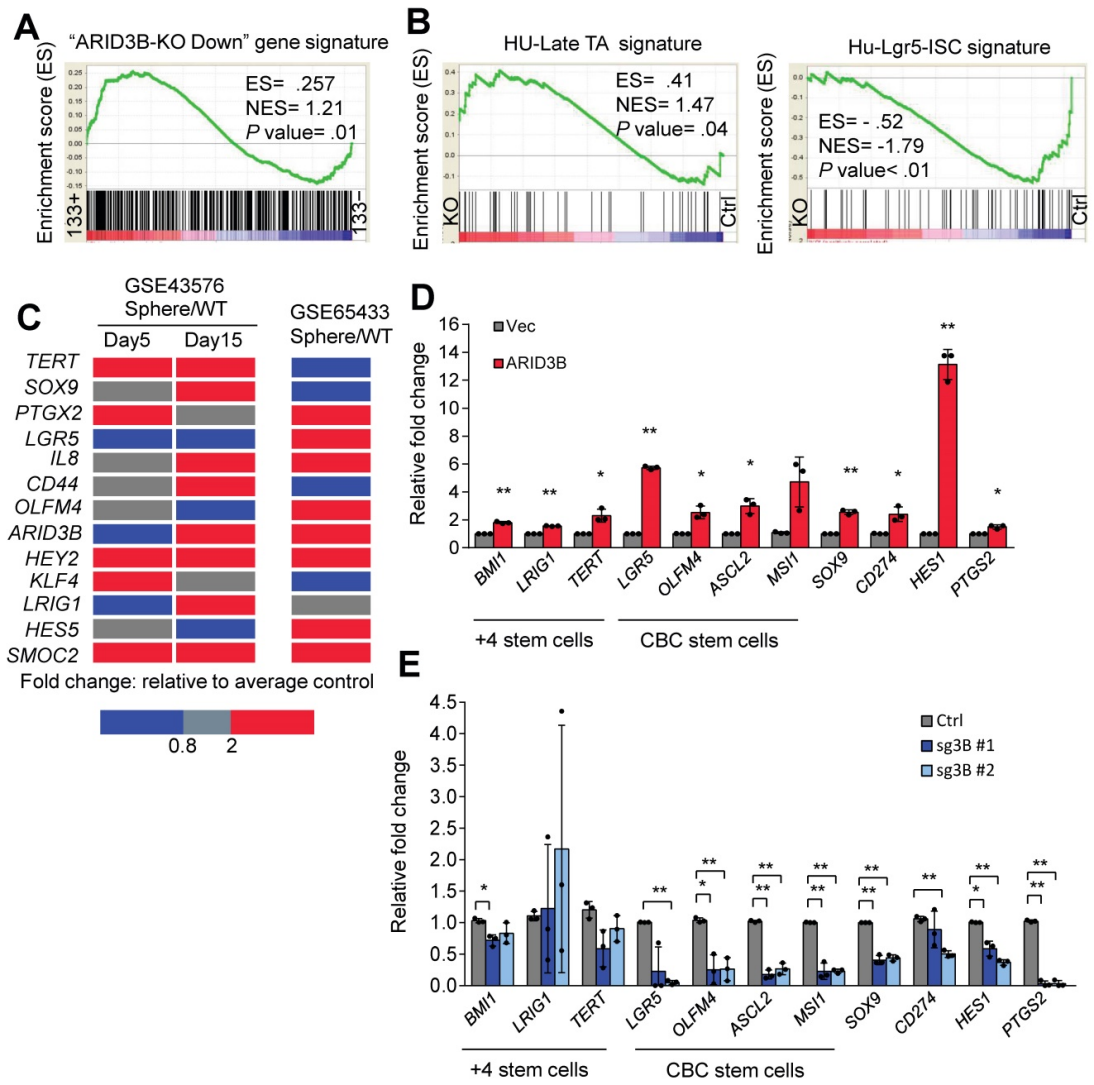
ARID3B expression is associated with the CRC stem-like gene signature. **A** GSEA shows the positive correlation between the ARID3B-regulated signature and the CD133+ gene expression profile of CRC patients.** B** GSEA for the correlation between the late transient amplifying cell (Late-TA)/Lgr5+intestinal stem cell (ISC) signature and the ARID3B-regulated gene profile in HCT-15 cells receiving CRISPR/Cas9 for depleting ARID3B vs. the control. **C** A heatmap shows the expression of ARID3B and colorectal stemness genes in sphere-derived cancer stem cells versus the parental HT-29 cells in GSE65433. In another independent dataset of GSE43579, ARID3B expression was not increased on day 5 with sphere culture but was enhanced with continued cultivation under spheroid conditions. Similar results were also shown for other ISC genes on day 15. Red, upregulation; blue, downregulation. **D** RT-qPCR analysis of the expression of ISC genes in HT-29 cells infected with ARID3B vs. a control vector (Vec). Data represent the mean ± S.D. n=3 independent experiments (each experiment contained two technical replicates). **E** RT-qPCR for analyzing the expression of ISC genes in HCT15 cells receiving CRISPR/Cas9 for depleting ARID3B (sg3B) vs. the control. Data represent the mean ± S.D. n=3 independent experiments (each experiment contained two technical replicates). **p < 0.01; *p < 0.05. For (A) and (B), ARID3B-KO Down, the genes downregulated ≥ 2.6-fold in ARID3B knockout cells. ES, enrichment score. NES, normalized enrichment score. See also Figure S2, Table S6.

We previously showed that ARID3B regulates the expression of pluripotency genes to enhance the stemness of head and neck cancer 36. Interestingly, the expression levels of these pluripotency genes were not affected by ARID3B in CRC cells (Figure S2G). We therefore hypothesized that ARID3B-regulated target genes are cell-type specific, and the understanding of the molecular context will extend our knowledge of how ARID3B regulates cancer stemness. A GSEA was performed to confirm the correlation between the ARID3B-regulated gene signature (Table S6) and CRCSCs. GSEA showed that the ARID3B-regulated signature was highly associated with CD133^+^ cells from CRC patient samples (Figure 2A). The ARID3B signature was also correlated with the presence of adenomatous polyps during colon tumorigenesis compared to that of nontumor tissues (Figure S2H). The Lgr5^+^ intestinal stem cell (ISC) signature is associated with CRCSCs, whereas the late transient amplifying cell (TA) signature has the opposite pattern 45. Here, we correlated the Lgr5^+^ ISC signature and late TA signature with the ARID3B-regulated signature in CRC. The Lgr5^+^ ISC signature was significantly associated with the ARID3B-regulated signature, whereas the late TA signature showed the opposite pattern (Figure 2B and Figure S2I).

We next validated the impact of ARID3B expression on key ISC genes. Upregulation of ARID3B, as well as the other CRC-specific stemness genes, was noted in the HT-29-derived tumorsphere from two independent datasets (Figure 2C). Furthermore, overexpression of ARID3B in HT-29 cells upregulated ISC genes, including *LGR5*,* OLFM4*, *ASCL2*, *MSI1*, and *SOX9,* and Notch pathway target genes, such as *HES1* and *PTGS2*; however, knockout of ARID3B in HCT-15 cells suppressed most of these genes (Figure 2D-E). Thus, ARID3B regulates ISC genes and promotes the stem-like features of CRC.

**Fig 3:**
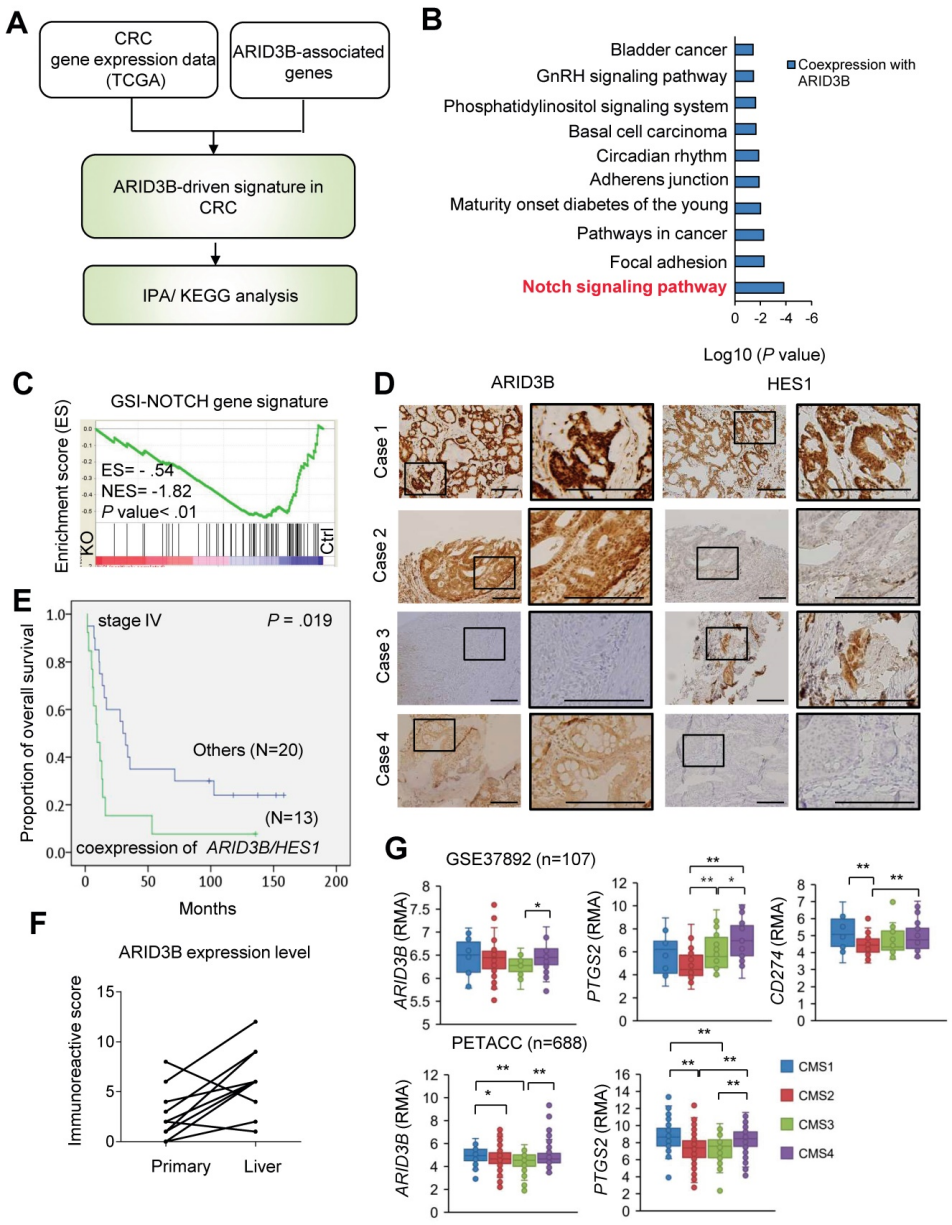
ARID3B correlates with the Notch pathway and PD-L1 expression in CRC patients. **A** flowchart for mining the key ARID3B-regulated pathways in CRC. **B** KEGG pathway analysis shows that the Notch signaling pathway was most significantly associated with ARID3B-driven signatures.** C** GSEA shows the negative correlation between the Notch signature (GSI-NOTCH) and the ARID3B-regulated gene profile in the HCT-15 cells receiving CRISPR/Cas9 for depleting ARID3B (KO) vs. the control (Ctrl). ES, enrichment score. NES, normalized enrichment score.** D** Representative immunohistochemical staining results of ARID3B and HES1 in CRC samples. Case 1, ARID3B^high^HES1^high^; case 2, ARID3B^high^HES1^low^; case 3, ARID3B^low^HES1^high^; case 4, ARID3B^low^HES1^low^. Scale bar=200 μm.** E** Kaplan-Meier analysis of the overall survival in 33 stage IV CRC patients with coexpression of ARID3B and HES1 shows a worse prognosis.** F** The immunoreactive score shows ARID3B expression enhanced in the metastatic liver sites in 15 pairs of matched primary-liver metastatic CRC samples. p=0.003, Wilcoxon signed-rank test.** G** Analysis of the expression pattern of *ARID3B*, *PTGS2*, and *CD274* in different CMS subtypes. The data were obtained from GSE37892 (n=107) and PETACC (n=688). **p < 0.01; *p < 0.05. See also Figure S3, Tables S7-9.

**ARID3B correlates with Notch pathway activation and PD-L1 expression in CRC.** We next narrowed down the key pathway(s) driven by ARID3B in CRC samples. First, we analyzed the overlapping genes of the CRC-expressed genes and the ARID3B-associated genes from The Cancer Genome Atlas (TCGA) database of human CRC 46 to identify the key pathway(s) involving these genes. Figure 3A illustrates the strategy for determining these key pathway(s), and the analyzed pathways are detailed in Table S7 and Figure 3B*.* The Notch pathway was the most prominent pathway associated with ARID3B (Figure 3B). The Notch pathway has an evolutionarily conserved role in cell fate and regulation of stem cell behavior 47,48,49. In CRC, Notch signaling is pivotal for generating and maintaining CSCs 50. We next determined the involvement of ARID3B in the Notch pathway in CRC. GSEA supported the association between the ARID3B-regulated signature (see Table S6) and the GSI-NOTCH gene set (genes that were downregulated by gamma secretase inhibitor treatment 42) (Figure 3C). A significant association between *ARID3B* and *HES1*, a primary downstream target of the Notch pathway 51, 52, was also found (Figure S3A). Next, we validated the association between the expression of ARID3B and HES1 expression in 130 samples from colon cancer patients. The demographics of these patients are shown in Table S8. The results for high versus low expression levels of ARID3B/HES1 are illustrated in Figure S3B, and representative patients for the different expression patterns of ARID3B/HES1 are shown in Figure 3*D*. Although the coexpression of ARID3B and HES1 did not have a significant impact on the overall survival in all patients (Figure S3C), a subgroup analysis demonstrated the prognostic impact of the coexpression of ARID3B and HES1 in stage IV CRC patients (the 3-year survival rates, 15.4% vs. 35.0%, p = 0.019) (Figure 3E). Analyzing an independent cohort from the public database (GSE12945) also revealed a trend of worse outcomes in CRC patients with coexpression of ARID3B and HES1 (Figure S3D). Furthermore, there was significantly increased expression of ARID3B in metastatic liver tumors compared with primary tumors (see Table S9 for patient demographics) (Figure 3F).

We next examined the expression of *ARID3B* and related genes in different molecular subtypes of CRC. Two independent public databases were retrieved to analyze the expression of *ARID3B* and *PTGS2*, a Notch target gene that encodes COX-2 53, in four different subtypes categorized by consensus molecular subtype (CMS) classification 54. We also examined the expression of *CD274* (which encodes PD-L1) in different CMS subtypes because the increased expression of PD-L1 has been noted in CSCs for eliciting immune evasion 22, 23. Higher expression of *ARID3B* was observed, particularly in CMS4, which is the subtype with the CSC signature 54, compared with that in the other subtypes. Interestingly, a higher level of *PTGS2* and *CD274* was also shown in CMS4 (Figure 3G). This intriguing result implies that the CSC signature-predominant CMS4 subgroup may have elevated ARID3B and PD-L1 expression and an activated Notch pathway. Further analyses of the CRC samples from TCGA database 46 demonstrated a significant association between *CD274* and *ARID3B*, *PTGS2* and *ARID3B*, and *PTGS2* and* CD274* (Figure 3SE). Together, the results indicate that ARID3B is associated with the activation of the Notch pathway and expression of PD-L1 in CRC.

**Fig 4:**
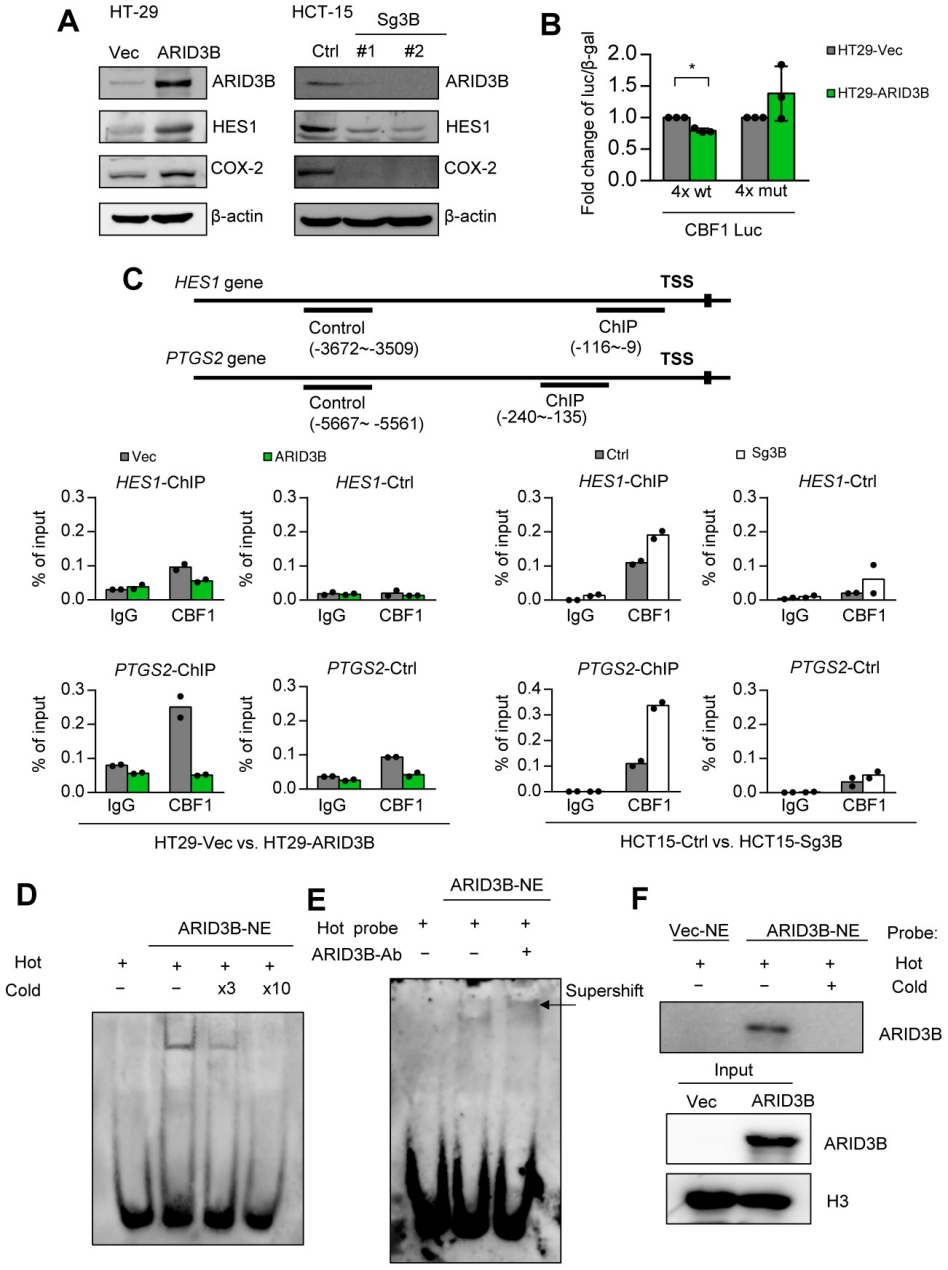
ARID3B binds to the CBF1 conserved binding site on the regulatory region of target genes.** A** Western blot shows the expression of HES1, and COX-2 was positively correlated with the expression of ARID3B in the HT-29 cells stably infected with ARID3B (HT29-ARID3B) versus a control vector (HT29-Vec) (left) or the HCT-15 cells depleted of ARID3B by CRISPR/Cas9 (HCT15-sg3B) versus the control (HCT15-Ctrl) (right). #1 and #2 represent two subclones. β-actin was used as a loading control.** B** Luciferase reporter assay of the HT-29 cells cotransfected with ARID3B expression plasmid/control vector, wild-type/mutant CBF1 reporter construct, and pCBV-β-gal. Data represent the mean ± SD. n = 3 independent experiments (each experiment contains two technical replicates). *p < 0.05.** C** ChIP assay. Upper, the schema showing the regulatory regions of *HES1* and *PTGS2* and the ChIP/control primers for the experiment. Lower: quantitative ChIP for analyzing the enrichment of CBF1 at the *HES1* (upper) and *PTGS2* (lower) promoters in HT29-ARID3B versus HT29-vector control (HT29-Vec) and HCT15-sg3B versus HCT15-Ctrl cells. One representative experiment of three independent experiments is shown. **D** Electrophoretic mobility shift assay (EMSA). The unlabeled probe was added at 3-fold (lane 3) or 10-fold (lane 4) concentrations for the competitive binding of the labeled probe. **E** EMSA and supershift assay. Left: Nuclear extracts (NE) from the HT29-ARID3B cells were incubated with the biotin-labeled probe with the 3xCBF1 conserved binding sequence (lane 2). The addition of the anti-ARID3B antibody resulted in a supershifted band (the arrow indicates the band in lane 3). No protein extract was added to lane 1.** F** Pull-down assay. Nuclear extracts from HT29-Vec or HT29-ARID3B were incubated with the biotin-labeled probe with the 3xCBF1 conserved binding sequence (lanes 1 and 2), and unlabeled oligonucleotides were added at a onefold concentration (lane 3). The extracts bound to the biotin-labeled probe bound were pulled down and analyzed by an anti-ARID3B antibody. H3 was a loading control. See also Figure S4.

**ARID3B regulates Notch target genes through a Notch intracellular domain-independent mechanism.** We next elucidated the mechanism of ARID3B-regulated Notch target genes in CRC. Two major Notch target genes, HES1 and COX-2, were selected as the model genes owing to their significance in cancer stemness 55, 56. We first validated the impact of ARID3B on the expression of these genes. The ectopic expression of ARID3B in HT-29 cells increased the expression of HES1 and COX-2, whereas the depletion of ARID3B by CRISPR-Cas9 in HCT-15 cells downregulated HES1 and COX-2 (Figure 4A). The canonical Notch pathway is activated by ligand-mediated proteolytic cleavage of membranous Notch receptors to release the Notch intracellular domain (NICD). The NICD enters the nucleus and acts as a coactivator together with other DNA-binding proteins, such as CBF1, to activate the transcription of target genes 57. We examined whether ARID3B upregulates the NICD in CRC cells. Surprisingly, the manipulation of ARID3B did not change the NICD level (Figure S4A). A promoter activity assay of HT-29 cells showed that ARID3B repressed the activity of the reporter construct, which contains four repeats of the binding sites for the NICD-interacting transcription factor CBF1. Mutating the binding sites abrogated the effect (Figure 4B). Analysis of the sequences showed a high similarity between the putative CBF1 and ARID3B binding motifs 58, and the motif on the promoter of *HES1* and *PTGS2* was highly conserved among different species (Figure S4B), indicating that ARID3B may compete with CBF1 for the conserved binding sites.

Next, we tested whether ARID3B interacts with the NICD to determine the effect of ARID3B on the occupancy of CBF1 at target genes. A coimmunoprecipitation assay showed that ARID3B was not coprecipitated with the NICD (Figure S4C). A ChIP assay showed a decreased enrichment of CBF1 at the promoters of *HES1* and *PTGS2* upon expression of ARID3B in HT-29 cells, whereas knockout of ARID3B in HCT-15 cells increased the binding of CBF1 to the *HES1* and *PTGS2* promoters (Figure 4C and Figure S4D). Furthermore, an electrophoretic mobility shift assay (EMSA) demonstrated the direct binding of ARID3B to a biotin-labeled probe containing three repeats of the CBF1 binding sequence. Increased ARID3B binding was observed after incubating nuclear extracts from ARID3B-overexpressing HT-29 cells with CBF1 binding site-containing oligonucleotides, and the competition for ARID3B binding by the unlabeled probe abolished the shifted band (Figure 4D and Figure S4E). A supershifted band was noted after adding an anti-ARID3B-specific antibody to the nuclear extracts of ARID3B-infected HT-29 cells (Figure 4E). The analysis of nuclear proteins bound to the biotin-labeled probe showed that ectopic expression of ARID3B increased the binding of ARID3B to the labeled probe, and the addition of the unlabeled probe abrogated this binding (Figure 4F). In summary, the above results indicate that ARID3B competes with CBF1 to bind to the consensus motif on the regulatory region of Notch target genes through an NICD-independent mechanism.

**Fig 5:**
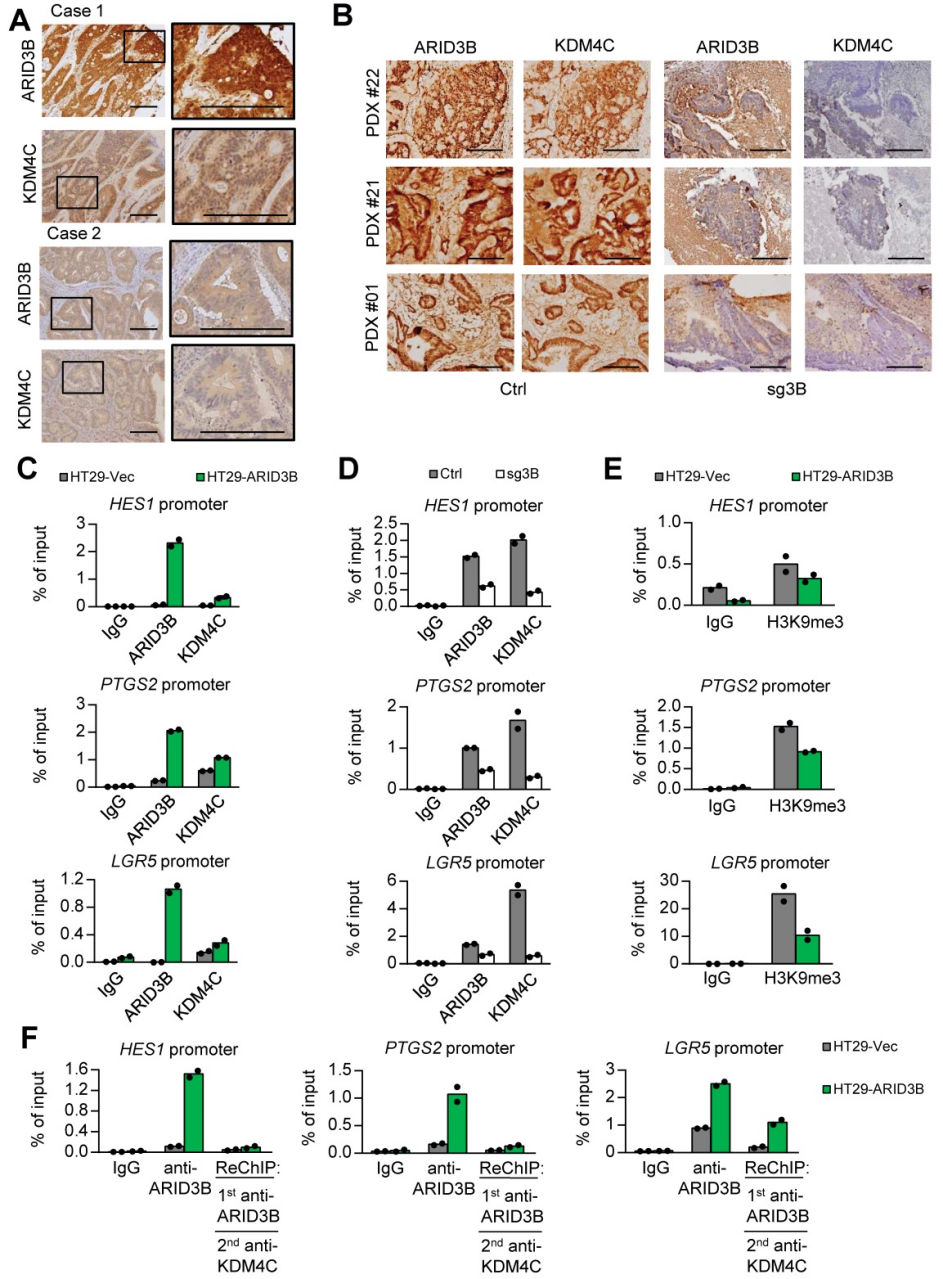
ARID3B recruits KDM4C for demethylating H3K9me3 at target genes. **A** Representative immunohistochemical staining results of ARID3B and KDM4C in CRC samples. Case 1, ARID3B^high^KDM4C^high^; case 2, ARID3B^low^KDM4C^low^. Scale bar=200 μm. **B** Representative images showing the expression of ARID3B and KDM4C in PDXs. Scale=200 µm. **C** ChIP shows the occupancy of ARID3B and KDM4C on the regulatory region of target genes in HT29-ARID3B vs. HT29-vector control (HT29-Vec). Signals amplified by the ChIP primers. One representative experiment of three independent experiments is shown. **D** ChIP shows the occupancy of ARID3B and KDM4C on the regulatory region of the target gene in HCT15-sg3B versus HCT15-Ctrl. Signals amplified by the ChIP primers. One representative experiment of three independent experiments is shown. **E** ChIP for analyzing the enrichment of H3K9me3 on the regulatory region of target genes in HT29-ARID3B vs. HT29-vector control (HT29-Vec) cells is shown. Signals amplified by the ChIP primers. One representative experiment of three independent experiments is shown.** F** Sequential ChIP for analyzing the co-occupancy of ARID3B and KDM4C on the promoters of target genes in HT29-ARID3B vs. HT29-vector control (HT29-Vec). One representative experiment of three independent experiments is shown. See also Figure S5, Tables S10-11.

**ARID3B recruits KDM4C for histone modification of target genes.** Because ARID3B binds to the regulatory region of Notch target genes through an NICD-independent mechanism in CRC cells (Figure 4), we examined whether ARID3B regulates Notch target genes through other known NICD/CBF1-independent pathways, such as β-catenin 59, GLI2 60, and phospho-JNK 61. However, the expression of ARID3B did not have a significant impact on these factors (Figure S5A-B). We previously demonstrated that in head and neck cancer cells, ARID3B forms a complex with ARID3A. The complex recruits KDM4C to the promoter of stemness factors to activate their transcription through demethylation of histone 3 lysine 9 trimethylation (H3K9me3) 36. Here, we analyzed the association between the expression of ARID3B, KDM4C, and HES1 in the study cohort. Representative KDM4C high versus low expression levels are shown in Figure S5C, and different patterns of ARID3B/KDM4C expression from representative patients are illustrated in Figure 5A. HES1 expression was significantly associated with both ARID3B and KDM4C (p= 0.028 and 0.007, respectively; Table S10). Analysis of the PDX samples also supports this notion: in the control group, ARID3B colocalized with KDM4C; in contrast, the depletion of ARID3B expression in PDXs dispersed KDM4C (Figure 5B), indicating that depletion of ARID3B in PDXs abolished the recruitment of KDM4C. Since ARID3A is another component in this complex in head and neck cancer stem cells 36, we also analyzed the association between ARID3A and other molecules in CRC samples. However, the expression of ARID3A was not associated with ARID3B and HES1 (Table S11). These results imply that for activation of Notch target genes in CRC, the formation of the ARID3A-ARID3B complex may not be as important as for the activation of pluripotency genes in head and neck cancer.

We next uncovered the mechanism of how ARID3B regulates ISC and Notch target genes in CRC. For Notch target genes (*HES1* and *PTGS2*), the CBF1-binding motif, which is also the presumptive binding motif for ARID3B, was analyzed. For ISC genes (*LGR5*, *MSI1*, and *SOX9*), the presumptive ARID3B binding region, i.e., the AT-rich region, was examined. Overexpression of ARID3B increased the recruitment of KDM4C to the regulatory region of Notch target genes and ISC genes (Figure 5C and Figure S5D). Knockout of ARID3B dissociated KDM4C from the regulatory region (Figure 5D and Figure S5D). Ectopic ARID3B reduced the enrichment of H3K9me3 (Figure 5E and Figure S5E). A sequential ChIP assay confirmed the co-occupancy of ARID3B and KDM4C on the regulatory region of target genes in ARID3B-overexpressing HT-29 cells (Figure 5F and Figure S5F). Interestingly, the sequential ChIP experiment also indicated that ARID3A did not show co-occupation with ARID3B on the regulatory region of target genes (Figure S5E), which is consistent with the findings in the clinical sample analyses. Altogether, these results suggest that ARID3B recruits KDM4C to the regulatory region of target genes, leading to reduced H3K9me3 and gene expression levels.

**Fig 6:**
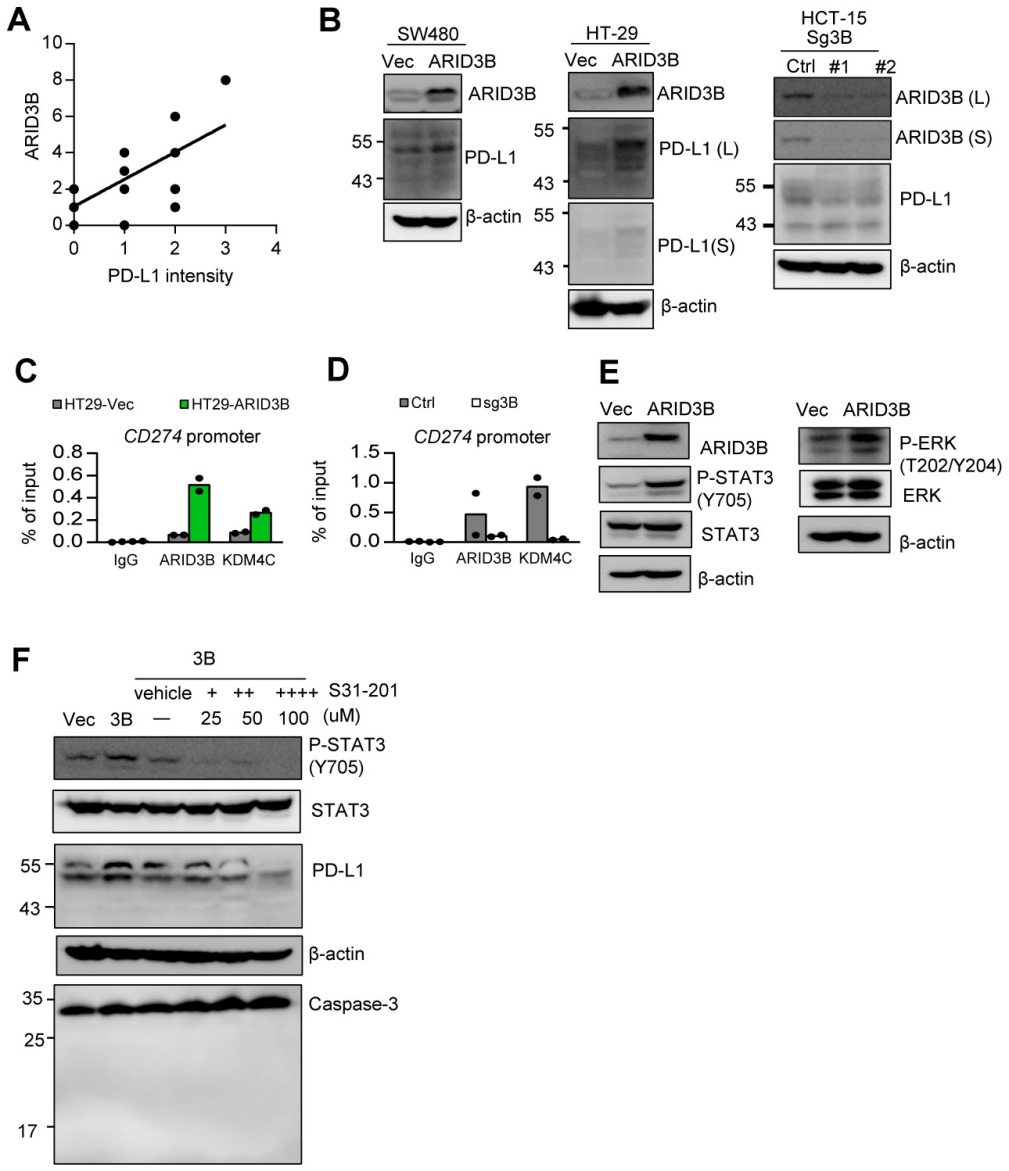
ARID3B controls PD-L1 expression through epigenetic regulation and STAT3-mediated activation. **A** A scattering plot presenting the positive correlation between the immunoreactive score of ARID3B and PD-L1 intensity in 15 CRC patient samples. **B** Western blot of PD-L1 expression in the SW480 and HT-29 cells stably infected with ARID3B (SW480/HT29-ARID3B) versus a control vector (SW480/HT29-Vec) (left) and the HCT-15 cells depleted of ARID3B by CRISPR/Cas9 (HCT15-sg3B) versus control (HCT15-Ctrl) (right). #1 and #2 represent two subclones. L, long exposure; S, short exposure. β-actin was used as a loading control. **C** ChIP shows the occupancy of ARID3B and KDM4C on the regulatory region of *CD274* in HT29-ARID3B vs. HT29-vector control (HT29-Vec) (left) and HCT-15 cells receiving CRISPR/Cas9 for targeting ARID3B (sg3B) versus a control vector (Ctrl) (right). The representative data were from three independent experiments. **D** ChIP assay shows the occupancy of ARID3B and KDM4C on the regulatory region of *CD274* in HT29-ARID3B vs. HT29-vector control (HT29-Vec). The representative data were from three independent experiments. **E** Western blots indicated that the levels of Y705-phosphorylated STAT3 and T202/Y204-phosphorylated ERK were increased, whereas the total STAT3 and ERK remained unchanged in HT29-ARID3B vs. HT29-vector control (HT29-Vec). β-actin was used as a loading control.** F** Western blots showing that PD-L1 was suppressed by treatment with the indicated inhibitors with decreased Y705-phosphorylated STAT3 in HT29-ARID3B cells for 24 h. Western blots showing full-length or cleaved caspase 3; the inhibitor did not induce cell apoptosis during the assay. See also Figure S6, Table S12.

**ARID3B regulates PD-L1 expression in CRC cells.** Our analysis of TCGA data indicated that both* ARID3B* and* CD274* were enriched in the CMS4 subtype (Figure 3G), and the expression of* CD274* was associated with *ARID3B* (Figure S3E). We wondered whether ARID3B participated in the process of enhanced PD-L1 expression in CRC. We first examined the association between ARID3B and PD-L1 in CRC patient samples. Representative PD-L1 expression levels for scoring are shown in Figure S6A. A significant positive correlation between ARID3B and PD-L1 was revealed (Figure 6A; Table S12). We next determined the impact of ARID3B manipulation on the expression of PD-L1. The results indicated that ectopic ARID3B expression increased PD-L1 expression in HT-29 and SW480 cells. The knockout of ARID3B in HCT-15 cells reduced PD-L1 expression (Figure 6B).

We next investigated the mechanism of ARID3B-regulated PD-L1 expression in CRC. First, we determined the direct regulation of PD-L1 by ARID3B. A ChIP assay showed that the ectopic expression of ARID3B in HT-29 cells increased the occupancy of ARID3B as well as KDM4C on the regulatory region of *CD274*. The depletion of ARID3B in HCT-15 cells reduced the binding of ARID3B and KDM4C to the regulatory region of *CD274* (Figure 6C). A sequential ChIP assay confirmed the co-occupancy of ARID3B and KDM4C on the regulatory region of *CD274* in the ARID3B-overexpressing HT-29 cells (Figure 6D). Next, we comprehensively examined the major signaling pathways regulating the expression of* CD274,* including the COX-2, MAPK, PI3K/AKT, and JAK/STAT pathways 62. Regarding the COX-2 pathway, a previous study showed that the COX-2-mediated pathway regulates PD-L1 expression in immune cells without a clear mechanism 63. However, the knockdown of COX-2 with two independent shRNA sequences in the HT29-ARID3B cells did not affect PD-L1 expression (Figure S6B), which is consistent with a report showing that COX-2 inhibition did not influence PD-L1 expression in lung cancer cells 64.

For the other reported pathways that regulate PD-L1, the overexpression of ARID3B increased the phosphorylation of ERK at T202/Y204 and STAT3 at Y705 (Figure 6E) but not the phosphorylation of AKT at S473 and STAT1 at Y701 (Figure S6C). Treatment with STAT3 inhibitors (S31-201) at a noncytotoxic concentration suppressed the ARID3B-induced PD-L1 expression (Figure 6F). However, the inhibition of MEK activity by a subcytotoxic dose of trametinib did not significantly influence PD-L1 (Figure S6D). Altogether, the above results indicate that ARID3B upregulates PD-L1 expression by directly binding to the regulatory region of *CD274* for epigenetic regulation as well by activating STAT3.

**Fig 7:**
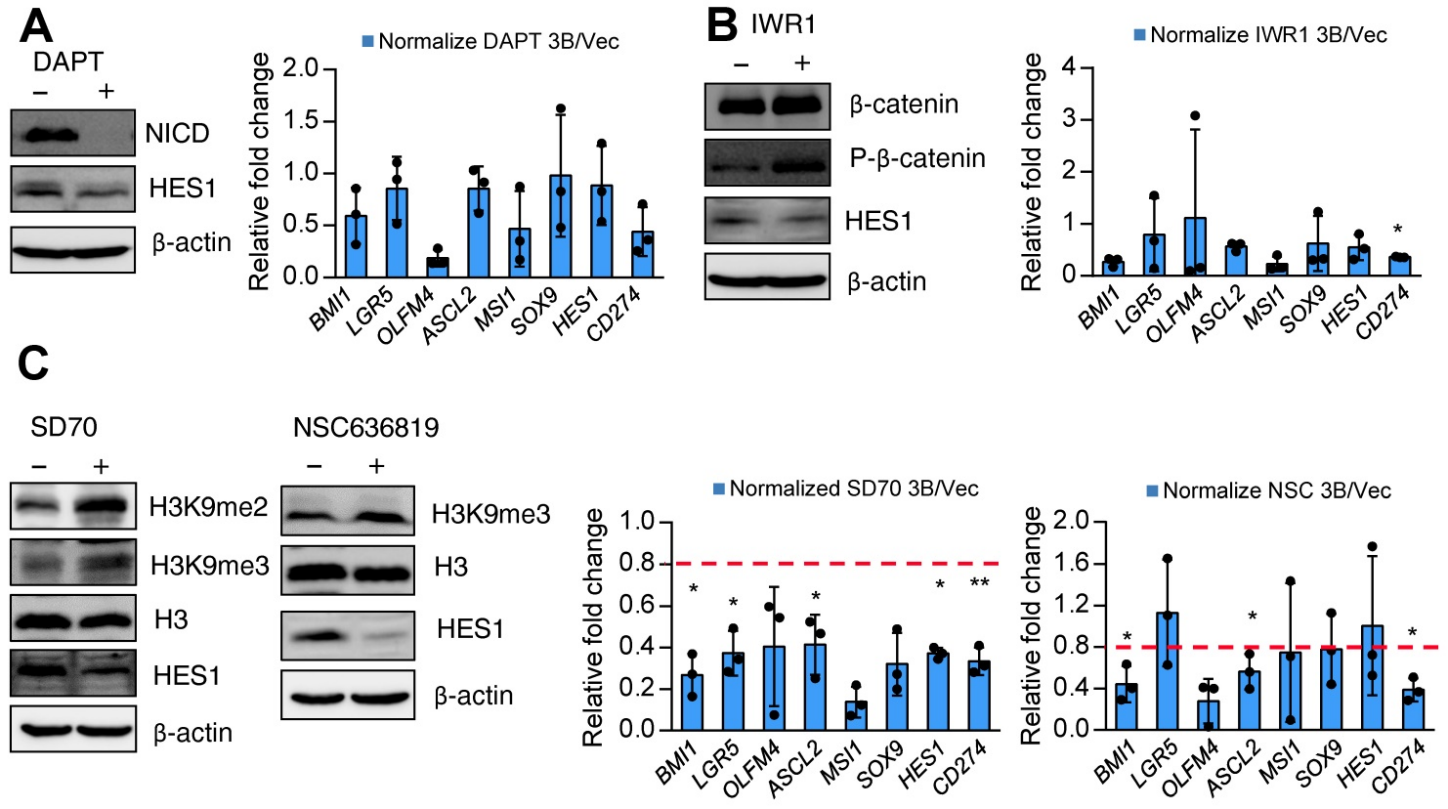
KDM4 inhibitors reverse ARID3B-induced target gene activation. **A-C** Left: Western blot for analyzing the expression of Notch intracellular domain (NICD) and HES1 in the HT29-ARID3B cells treated with the Notch inhibitor DAPT, Wnt inhibitor IWR1, and KDM4A/B inhibitor NSC636819 or KDM4C inhibitor SD70 for 24 h. The working concentration of DAPT was 16 μM, of IWAR1 was 64 μM, of WNT-59 was 4 μM, of SD70 was 16 μM, and of NSC636819 was 64 μM for 24 h. Among them, the SD70 inhibitor showed the most significant effect on suppressing the activation of ARID3B downstream targets. Right: RT-qPCR for analyzing the relative expression of intestinal stem cell genes and *CD274* in the above conditions. Data represent the mean ± SD. n = 3 independent experiments (each experiment contained two technical replicates). **p < 0.01. *p < 0.05. See also Figure S7.

**Pharmacological inhibition of KDM4 activity attenuates ARID3B-induced expression of target genes.** Finally, we sought a potential strategy for reversing ARID3B-mediated target gene activation in CRC. We first screened the effect of different inhibitors, including a canonical Notch pathway inhibitor (DAPT), Wnt pathway inhibitor (IWR1), KDM4C inhibitor (SD70), and KDM4A/B inhibitor (NSC636819), on suppressing the proliferation of ARID3B-overexpressing CRC cells. Since these drugs mainly target stemness genes, no significant differences were observed between the proliferation rates of the HT29-ARID3B and HT29-vector control cells after treatment with different inhibitors for 24 h (Figure S7A). We next examined the effect of these inhibitors on the expression of Notch target genes, ISC genes, and *CD274* in CRC cells. The suppression of NICD validated the pharmacological impact of DAPT, and an increased level of phospho-β-catenin confirmed the effect of IWR1. Inhibition of Notch activity by DAPT and suppression of Wnt activity by IWR1 mildly reduced the expression of HES1 but did not have a consistent and significant impact on the other downstream target genes (Figure 7A-B). For the KDM inhibitors, increased H3K9me3 was detected after treatment with the KDM4C inhibitor SD70 as well as the KDM4A/B inhibitor NSC636819 (Figure 7C, left panel). The complementary activities between the KDM4 family demethylases have been reported 65, and this notion was supported by an *in vitro* demethylation assay that demonstrated the suppression of the demethylase activity of KDM4C by the KDM4A/B inhibitor NSC636819 (Figure S7B). We assumed that inhibiting KDM4A/B also suppresses KDM4C activity and downregulates ARID3B-regulated target genes. Treatment with SD70 and NSC636819 both showed different extents of downregulation of the ARID3B-induced target genes, and SD70 had a more prominent effect (Figure 7C). The above data indicated that the pharmacological inhibition of KDM4 activity attenuates the expression of the ARID3B-induced Notch target genes, ISC genes, and* CD274* in CRC cells.

We summarize our findings in the graphic abstract. In CRC cells, ARID3B activates Notch target genes and ISC genes through an NICD-independent mechanism. ARID3B binds to the conserved motif of target genes and recruits KDM4C to demethylate H3K9me3, resulting in transcriptional activation of target genes. Activation of Notch target genes and ISC genes leads to the stem-like phenotype and increases PD-L1 in CSCs, which contributes to the immune evasion of CSCs. The pharmacological inhibition of KDM4 activity reverses ARID3B-mediated target gene activation.

## Discussion

In addition to the canonical Notch pathway that involves the ligand-induced cleavage of Notch to generate the NICD for transcriptional regulation 57, the noncanonical Notch pathway has been gradually uncovered. The most prominent example is the reciprocal regulation between the Notch and Wnt/β-catenin pathways. Notch-dependent regulation of β-catenin does not require ligand-dependent membrane cleavage of Notch. Instead, it requires Numb and lysosomal activity 59. In addition to β-catenin, the sonic hedgehog and JNK pathways can activate Notch targets 60. Here, we identified a novel noncanonical Notch pathway in CRC via ARID3B. Based on our results together with the previous findings of the canonical Notch pathway in CRCSCs 66, we herein suggest that both the canonical and noncanonical Notch pathways contribute to generating CRCSCs. Examining the expression of Notch receptor/ligand expression is not suitable to represent the stem cell features of CRC, and canonical Notch inhibitors (e.g., γ-secretase inhibitor) may not be sufficient to repress CRCSCs.

Accumulating evidence indicates that the expression of stem cell factors in CSCs is tissue specific. For example, the expression of pluripotency genes is associated with CSCs in head and neck cancers 36, 67, ISC gene expression is the key feature of CRCSCs 15, 16, 43, 66, and the hepatic stem cell gene signature was found in CSCs in liver cancer 68. However, the underlying mechanisms responsible for the tissue-specific CSC signature are unclear. There are two types of intestinal stem cells defined, cycling crypt base columnar cells (CBCs) and quiescent +4 cells 69. CBC stem cells confer intestinal renewal during homeostasis and are rapidly dividing, whereas +4 stem cells remain a nondividing and quiescent population of 'reserve' stem cells that coexist with CBC stem cells 70. Our data showed that the overexpression of ARID3B enhanced the ISC gene expression of both CBC and +4 stem cell markers 71. However, reduced ARID3B only significantly decreased the expression of the CBC stem cell markers. The results indicated that the CBC stemness factors might be the primary downstream targets of ARID3B. In this study, we demonstrated that ARID3B upregulates the Notch targets (HES1 and COX-2) and ISC genes in CRCSCs without affecting the expression of pluripotency factors. This finding is distinct from our previous study, which showed that ARID3B induces *POU5F1, NANOG,* and *SOX2* in head and neck CSCs 36. The differential mechanisms of ARID3B may explain the context dependency of CSC factors among different cancers.

The major obstacle for applying ICIs in CRC is that ICIs are effective only for patients with microsatellite instability (MSI), which is less than 15% of CRC patients 54. The CMS molecular classification of CRC suggests that MSI patients belong to the CMS1 subtype 9. However, among the other molecular subtypes of microsatellite-stable (MSS) CRCs, the most aggressive subtype is CMS4, which has the EMT signature 54, frequently associated with an advanced stage and worse survival 72, and is considered a “hot tumor” with immunosuppressive signaling. Here, we showed that ARID3B dominates the stem cell signature and PD-L1 expression in CRC, and the ARID3B-regulated signature may be a prevailing feature in the CMS4 subtype. This finding unveils a mechanism of immune evasion in CRCSCs. Clinically, together with the findings from a previous study showing that the CMS4 transcriptome contributes to acquired resistance to anti-EGFR treatment 73, these results indicate that customized targeting of the CMS4 subtype with ICIs may be considered to extend the application of ICIs in CRC.

In this study, we also aimed to develop strategies to reverse ARID3B-induced changes in CRCSCs. Treatment with a canonical Notch inhibitor and Wnt inhibitor did not influence the expression of target genes in the ARID3B-overexpressing CRC cells. In contrast, inhibition of KDM4 activity attenuated the ARID3B-induced target gene expression. We also noted that NSC636819, which was initially developed as a KDM4A/B inhibitor, could suppress the demethylation of H3K9me3/H3K9me2. The complementary effect between KDM4 family demethylases should be considered since they are structurally similar to each other and have similar target specificities and comparable enzymatic activities 74, 75. Since PD-L1 is known to be regulated by multiple factors, we therefore were interested in whether ARID3B coordinates with other factors to regulate PD-L1 expression. Our results showed that ARID3B enhances phosphorylation at STAT3 Y705 to regulate PD-L1 expression. A previous study showed that STAT3 is activated in the presence of Notch signaling, and HES1 interacts with STAT3 to promote STAT3 phosphorylation and downstream signaling 76. Based on our results, we concluded that ARID3B regulates PD-L1 signaling in CRC through two different manners: direct regulation or through the STAT3 pathway, which is potentially mediated by HES1-STAT3 crosstalk. Because ARID3B is not a druggable target, our findings suggest the manipulation of PD-L1 expression through STAT3 inhibitors. Together with the finding that ARID3B directly regulates PD-L1, the potential use of anti-PD1 therapy or STAT3 inhibitors to resume antitumor immunity in ARID3B-overexpressing CRC is worthy of further investigation.

In conclusion, our study reveals a previously unknown noncanonical Notch pathway driven by ARID3B in CRCSCs. We also identify the potential of applying ICIs in a specific molecular subtype of CRC. Epigenetic drugs reversing CSC features will be beneficial for the treatment of advanced CRC. These results provide valuable information for the future development of therapies against CRCSCs.

## Abbreviations

ARID3B: AT-rich interaction domain-containing protein 3B
ChIP: chromatin immunoprecipitation
CMS: consensus molecular subtype
CRC: colorectal cancer
CRCSC: colorectal cancer stem cell
CSC: cancer stem cell
EMSA: electrophoretic mobility shift assay
GSEA: Gene Set Enrichment Analysis
ICI: immune checkpoint inhibitor
IDLV: integrase-deficient lentiviral vectors
IHC: immunohistochemical staining
KDM4C: lysine-specific demethylase 4C
ISC: intestinal stem cell
mAb: monoclonal antibody
NICD: Notch intracellular domain
PD-1: programmed cell death protein-1
PD-L1: programmed death-ligand 1
PDX: patient-derived xenograft
TA: transient amplifying cells
TCGA: The Cancer Genome Atlas
