# Supplementary material for: Harnessing stemness and PD-L1 expression by AT-rich interaction domain-containing protein 3B in colorectal cancer
Source: Theranostics. 2020 May 15;10(14):6095–112. doi: 10.7150/thno.44147 (PMC7255042; doi:10.7150/thno.44147)
Supplement: Supplementary file 1 — Supplementary figures and tables. [file thnov10p6095s1.pdf]

Supplementary Figures

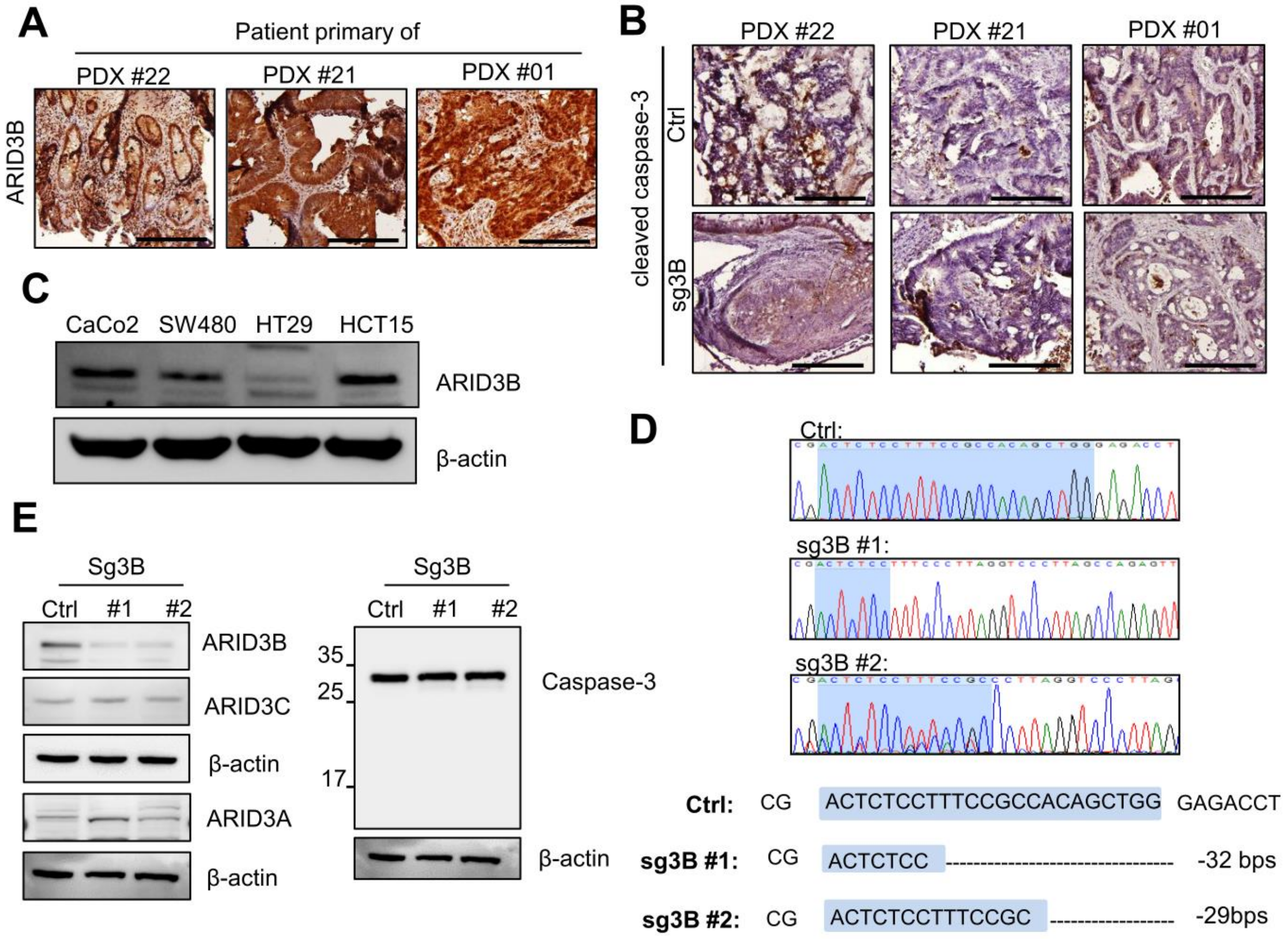

Figure S1. Expression profile and the generation of the ARID3B-knockout in CRC cell lines and PDXs. **A** IHC of ARID3B in the tumors of 3 CRC patients for generating PDXs. Scale bar=200μm. **B** Immunohistochemical staining of cleaved caspase-3 for indicating apoptosis in PDXs received the ARID3B knockout vector (sg3B) or a control vector (Ctrl) . Scale=200 μm. **C** Western blot shows the endogenous level ARID3B in four CRC cell lines CaCo2, SW480, HT29, and HCT15. β-actin was a loading control for western blot. **D** Sequencing results of the HCT-15 cells received CRISPR/Cas9 for depleting ARID3B (HCT15-sg3B) or control (HCT15-Ctrl). #1 and 2# represent two subclones. **E** Western blots of HCT-15 cells received CRISPR/Cas9 for depleting ARID3B (HCT15-sg3B) or control (HCT15-Ctrl). #1 and 2# represent two subclones. The expression of ARID3 family members was also examined to show the specificity of the sg3B sequence. The full length or cleaved Caspase 3 was the indicator for apoptosis, and the results showed that sg3B knockout did not induce the expression of cleaved Caspase 3.

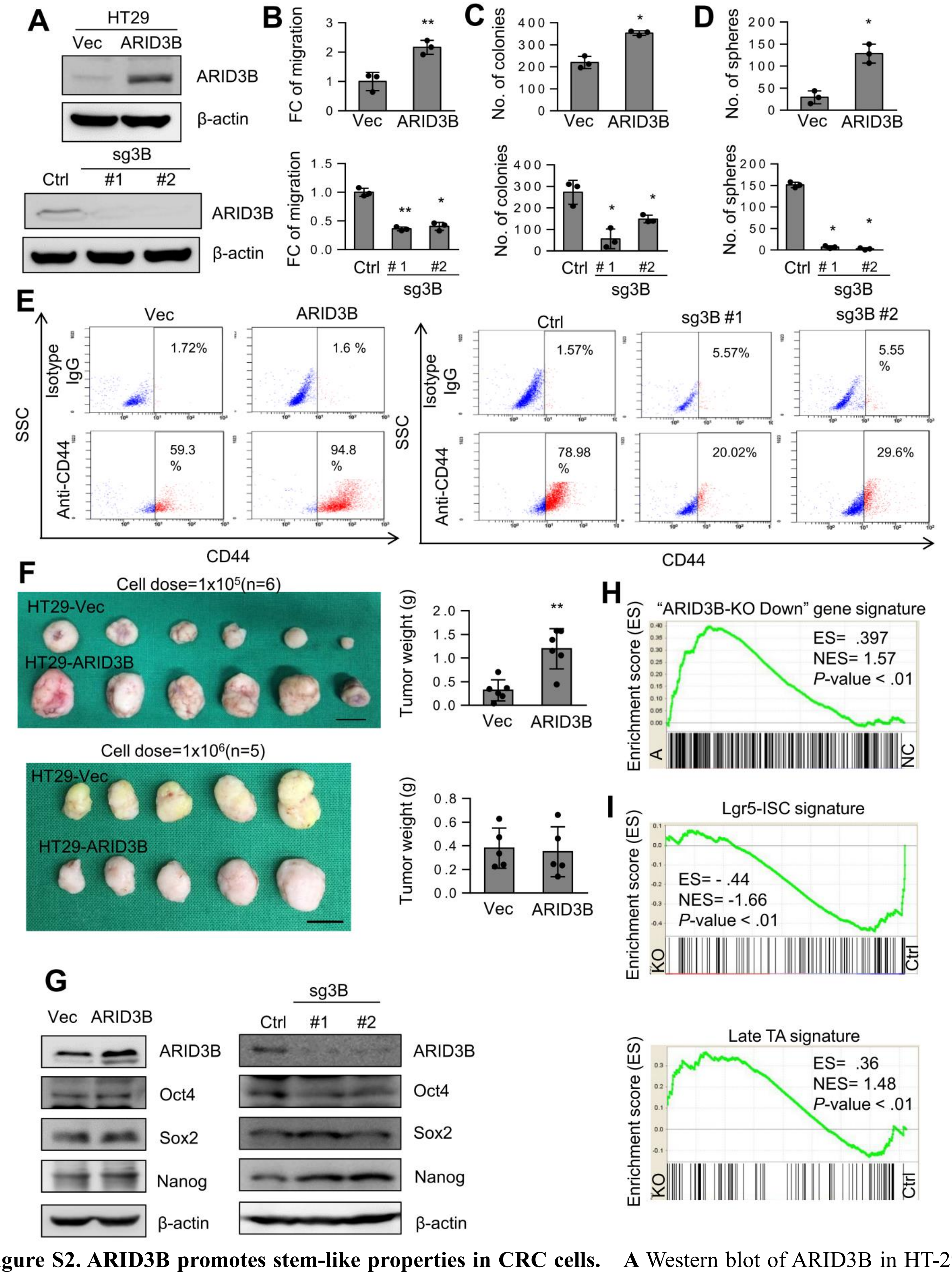

Figure S2. ARID3B promotes stem-like properties in CRC cells. **A** Western blot of ARID3B in HT-29 cells infected with an ARID3B expression vector vs. a control vector (Vec) (upper), and HCT-15 cells received CRISPR/Cas9 for depleting ARID3B vs. control (lower). #1 and 2# represent two subclones. β-actin

was a loading control. **B** Fold changes of migration in HT29-ARID3B vs. HT29-vector control (upper) and HCT15-sg3B vs. HCT15-Ctrl (lower). Data represent mean ± S.D. n=3 independent experiments (each experiment contains three technical replicates). *p<0.05. **C** Fold changes of the soft agar colony formation assay in HT29-ARID3B vs. HT29-Vec (upper) and HCT15-sg3B vs. HCT15-Ctrl (lower). Cell dose=5000/experiment. Data represent mean ± S.D. n=3 independent experiments (each experiment contains three technical replicates). *p<0.05. **D** Quantification of the spheroid formation assay. Upper: HT29-ARID3B vs. HT29-Vec. Lower: HCT15-sg3B vs. HCT15-Ctrl. Data represent mean ± S.D. n=3 independent experiments (each experiment contains three technical replicates). *p<0.05 **E** Flow cytometry of CD44 in HT29-ARID3B vs. HT29-Vec (left) and HCT15-sg3B vs. HCT15-Ctrl (right). The percentage of CD44-positive cells was displayed in the right upper quadrant of each panel. Isotype IgG was a control for flow cytometry. **F** Xenotransplantation assay. Upper: cell dose=$1x10^5$/each mouse. Lower: cell dose=$1x10^6$/each mouse. n=6 for each group. Scale bar=1cm. **G** Western blot of ARID3B, Oct4, Sox2, and Nanog in HT-29 cells stably infected with the ARID3B expression vector (HT29-ARID3B) versus a control vector (HT29-Vec)(left), and HCT-15 cells depleted ARID3B by CRISPR/Cas9 (HCT15-sg3B) versus control (HCT15-Ctrl)(right). β-actin was a loading control. **H** GSEA for analyzing the correlation between the ARID3B-regulated genes in HCT15-sg3B vs. HCT15-Ctrl and the tumorigenesis gene expression profile of CRC patients in different clinical status (GSE77953). ARID3B-KO Down, the genes downregulated ≥ 2.6 folds in ARID3B knockout cells. NC, normal colonic tissues; A, adenomas. ES, enrichment score. NES, normalized enrichment score. **I** GSEA for analyzing the correlation between the ARID3B-regulated genes in HCT15-sg3B vs. HCT15-Ctrl and Lgr5-intestinal stem cell (ISC) signature, and late transient amplifying (Late TA) signature. ARID3B-KO Down, the genes downregulated ≥ 2.6 folds in ARID3B knockout cells. ES, enrichment score. NES, normalized enrichment score.

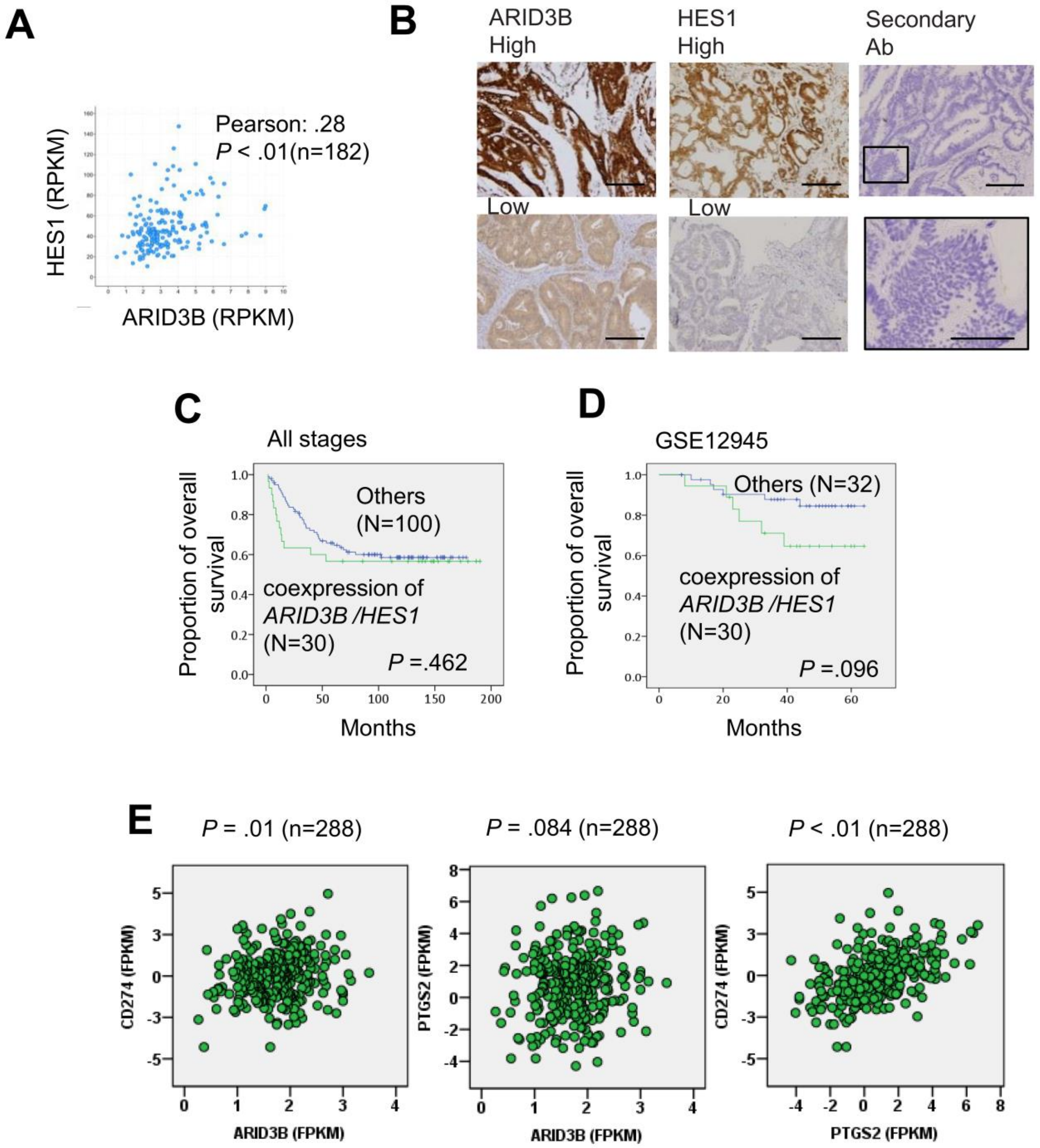

Figure S3. ARID3B expression correlates with HES1 and CD274. **A** A scattering plot illustrates the positive correlation between the expression of ARID3B and HES1 in the CRC samples obtained from TCGA (n=182). The data was obtained from the public database of Nature 2012;487:330-337. **B** Representative images of immunohistochemical staining for defining the results of ARID3B and HES1 in 130 CRC patient samples. Scale bar=200μm; for the magnified region, scale bar=100μm. **C** Kaplan-Meier analysis of overall survival in 130 CRC patients in all stages shows with or without co-expression of ARID3B and HES1. **D** Kaplan-Meier analysis of overall survival in 62 CRC patients in all stages with or without co-expression of ARID3B and HES1. The data was obtained from GSE12945. **E** Scattering plots illustrate the correlation between the expression of ARID3B and CD274 (left), PTGS2 and ARID3B (middle), and CD274 and ARID3B (right) in CRC samples obtained from TCGA (n=288).

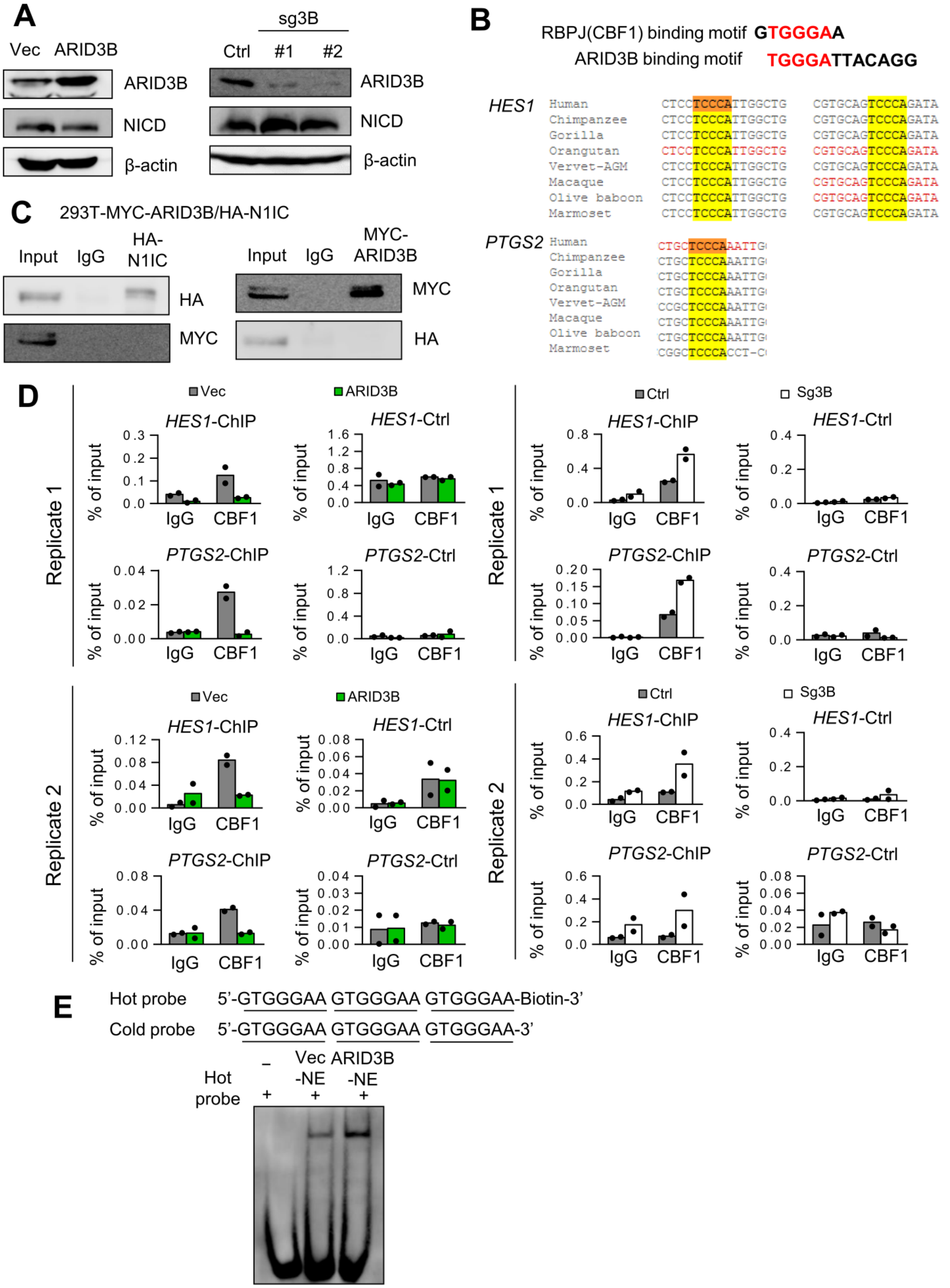
A
Vec ARID3B
ARID3B
NICD
β-actin
sg3B
Ctrl #1 #2
ARID3B
NICD
β-actin
B
RBPJ(CBF1) binding motif GTGGGAA
ARID3B binding motif TGGGATTACAGG
HES1
Human
Chimpanzee
Gorilla
Orangutan
Vervet-AGM
Macaque
Olive baboon
Marmoset
CTCCTCCCATTGGCTG
CGTGCAGTCCCAGATA
PTGS2
CTGCTCCCAAATTG
CCGCTCCCAAATTG
CGGCTCCCACCT-C
C
293T-MYC-ARID3B/HA-N1IC
Input IgG HA-N1IC
HA
MYC
Input IgG MYC-ARID3B
MYC
HA
D
Replicate 1
Replicate 2
Vec
ARID3B
Ctrl
Sg3B
HES1-ChIP
HES1-Ctrl
PTGS2-ChIP
PTGS2-Ctrl
% of input
IgG CBF1
E
Hot probe 5'-GTGGGAA GTGGGAA GTGGGAA-Biotin-3'
Cold probe 5'-GTGGGAA GTGGGAA GTGGGAA-3'
Hot probe
–
Vec -NE
ARID3B -NE
+ + +

Figure S4. ARID3B induces HES1 and COX-2 expression through an NICD-independent mechanism. **A** Western blot shows that manipulation of ARID3B does not affect NICD expression in both HT-29 cells stably infected with an ARID3B expression vector (HT29-ARID3B) versus a control vector (HT29-Vec) (left) and HCT15 cells depleted ARID3B by CRISPR/Cas9 (HCT15-sg3B) versus control (HCT15-Ctrl) (right). 1# and 2# represent two subclones. β-actin was a control. **B** The schema for showing the conserved binding regions of CBF1 and ARID3B on the regulatory region of HES1 and PTSG2. Upper: partial overlapping of the ARID3B binding motif predicted by MEME software with the CBF1 binding motif. Lower: alignment of the reverse complement of the CBF1/ARID3B binding motif on the regulatory region of HES1 and PTSG2 among different species. **C** Immunoprecipitation-western blot showing that MYC-tagged ARID3B does not physically interact with HA-tagged N1IC (NICD) in HEK-293T cells transfected with the indicated plasmids. **D** Quantitative ChIP for analyzing the enrichment of the regulatory region od CBF1 at HES1 (upper) and PTGS2 (lower) in HT29-ARID3B versus HT29-vector control (HT29-Vec), and HCT15-sg3B versus HCT15-Ctrl. These are another two independent biological replicates for Fig. 4c. **E** Electrophorectic mobility shift assay. Upper: the sequences of the probes. Lower: nuclear extracts (NE) from HT29-ARID3B or HT29-Vec cells were incubated with the biotin-labeled probe with 3xCBF1 conserved binding sequence.

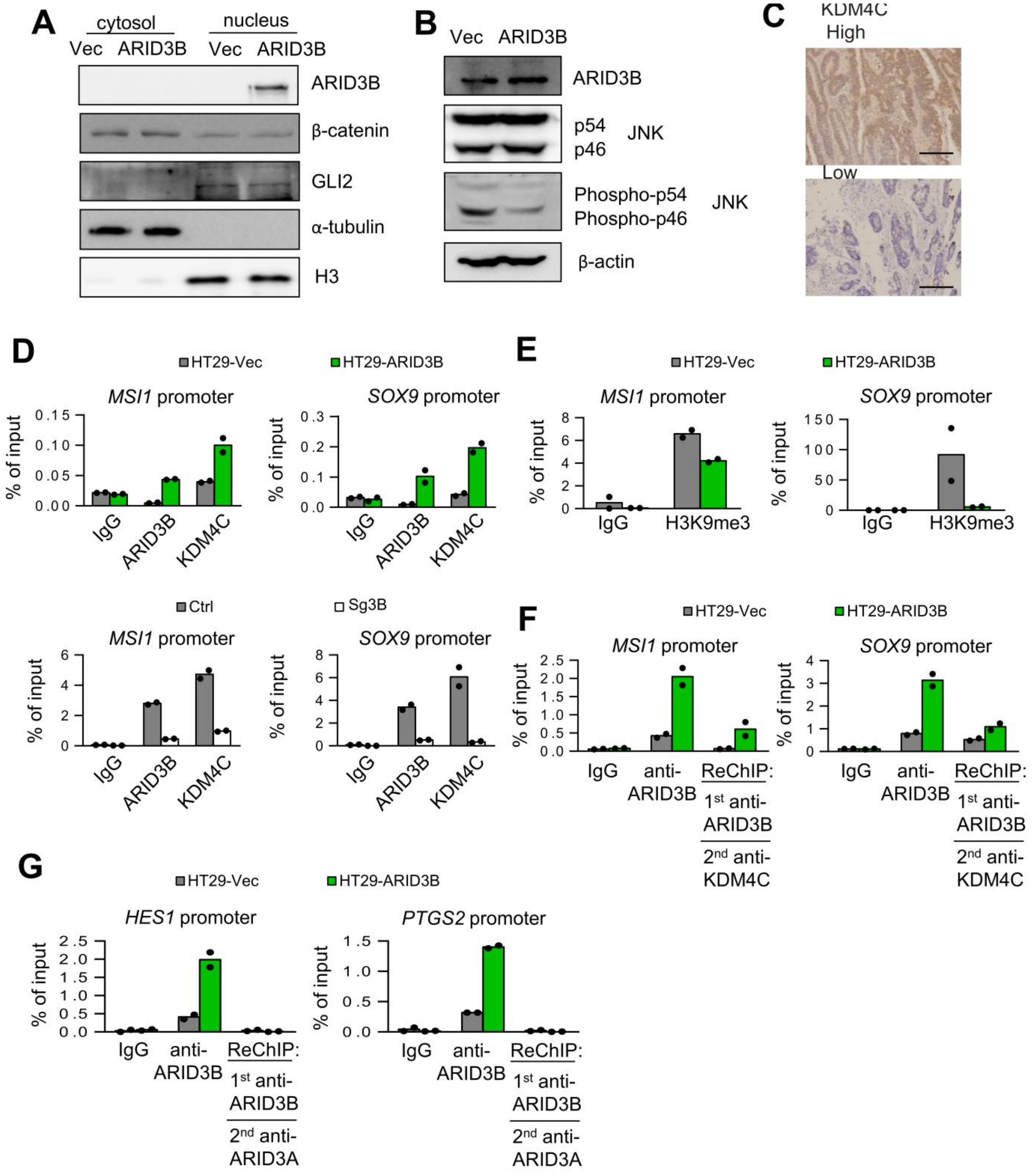

Figure S5. ARID3B activates downstream targets through the demethylation of H3K9me3. **A** Western blot shows the expression of ARID3B does not affect the expression and localization of GLI2, and β-catenin in the nucleus or cytosolic fraction of the HT-29 cells stably infected with an ARID3B expression vector (HT29-ARID3B) versus a control vector (HT29-Vec). α-tubulin was a loading control for cytosolic fraction, and H3 was a loading control for nucleus fraction. **B** Western blot of ARID3B, JNK and phosphorylated JNK in HT-29 cells stably infected with ARID3B (HT29-ARID3B) versus a control vector (HT29-Vec).**C** Representative images of immunohistochemistry for defining the staining results of KDM4C in 130 CRC patient samples. Scale bar=200μm. **D** ChIP assay. Left: ChIP results showing the occupancy of ARID3B and KDM4C on the regulatory region of target genes (MSI1 and SOX9) in HT29-ARID3B vs. HT29-vector control (HT29-Vec) or HCT15-sg3B versus HCT15-Ctrl. One representative experiment out of three independent

experiments is shown. **E** ChIP for analyzing the enrichment of H3K9me3 on the regulatory region of target genes (MSI1 and SOX9) in HT29-ARID3B vs. HT29-vector control (HT29-Vec) was shown. Signals amplified by the ChIP primers. One representative experiment out of three independent experiments is shown. **F** Sequential ChIP results show that the co-occupancy of ARID3B and KDM4C on the regulatory region of target genes (HES1 and PTGS2) in HT29-ARID3B vs. HT29-vector control (HT29-Vec). One representative experiment out of three independent experiments is shown. **G** Sequential ChIP results show that the co-occupancy of ARID3B and ARID3A on the regulatory region of target genes (HES1 and PTGS2) in HT29-ARID3B vs. HT29-vector control (HT29-Vec). Signals amplified by the ChIP primers. One representative experiment out of three independent experiments is shown.

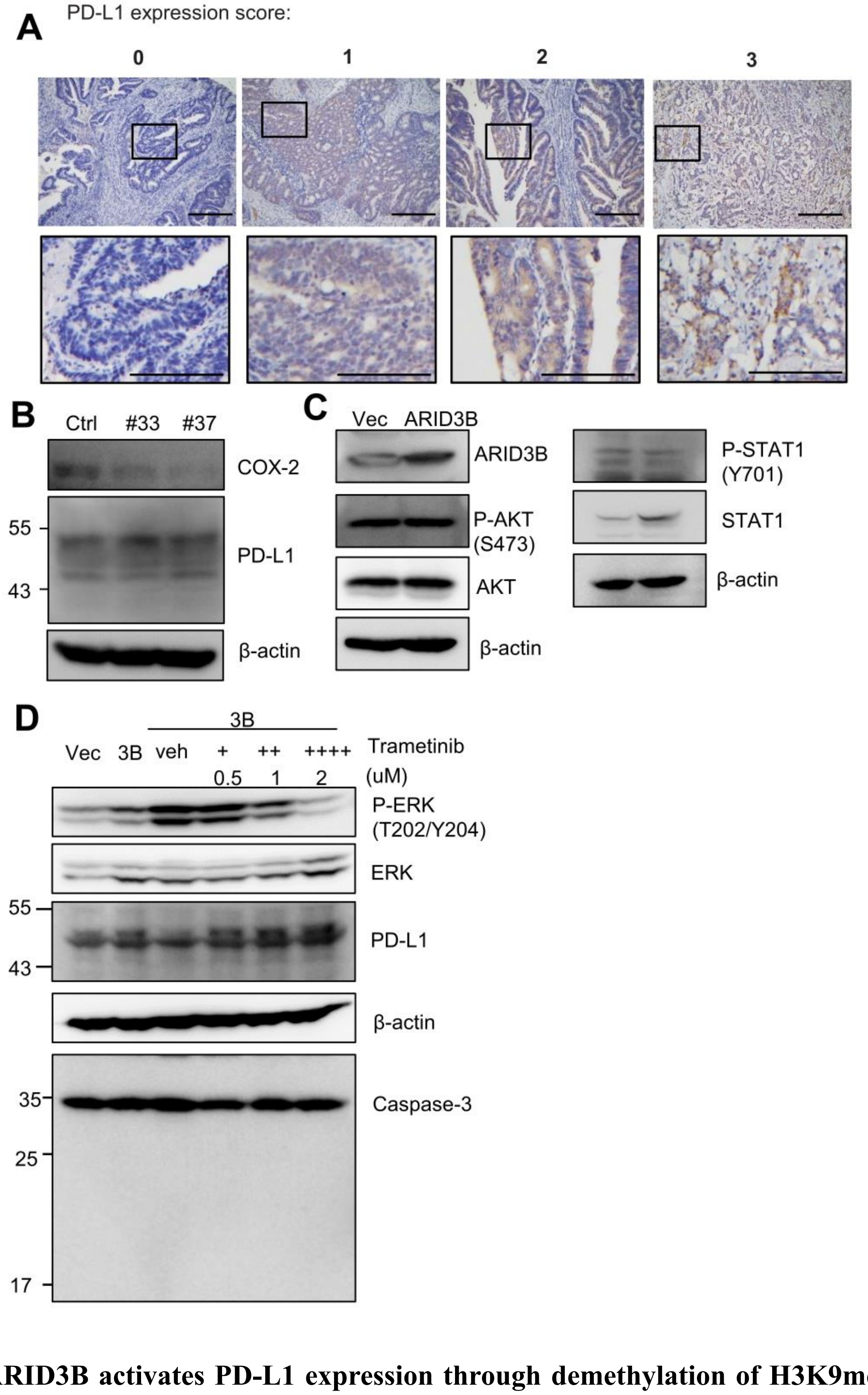

Figure S6. ARID3B activates PD-L1 expression through demethylation of H3K9me3 and STAT3. A Representative photos of immunohistochemistry staining of PD-L1 in CRC samples. PD-L1 expression score: 0, absent; 1, weak; 2, moderate; 3, strong. Scale bar: 200μm for upper representative photos, 100μm of lower representative photos. **B** Western blot of COX-2 and PD-L1 in HT29-ARID3B cells receiving shRNAs specific to PTGS2 (two independent shPTGS2 sequences; #33, #37) or a scrambled sequence (Ctrl).**C** Western blots show the level of S473-phosphorylated AKT, total AKT, Y701-phosphorylated STAT1, total STAT1, Y727-phosphorylated STAT3, and total STAT3 in HT29-ARID3B vs. HT29-vector control (HT29-Vec). β-actin was a loading control. **D** Western blots show the PD-L1, T202/Y204-phosphorylated ERK, ERK, and caspase-3 in HT29-ARID3B cells under the treatment of the ERK inhibitor Trametinib with the 0.5, 1, 2 μM for 24 h.

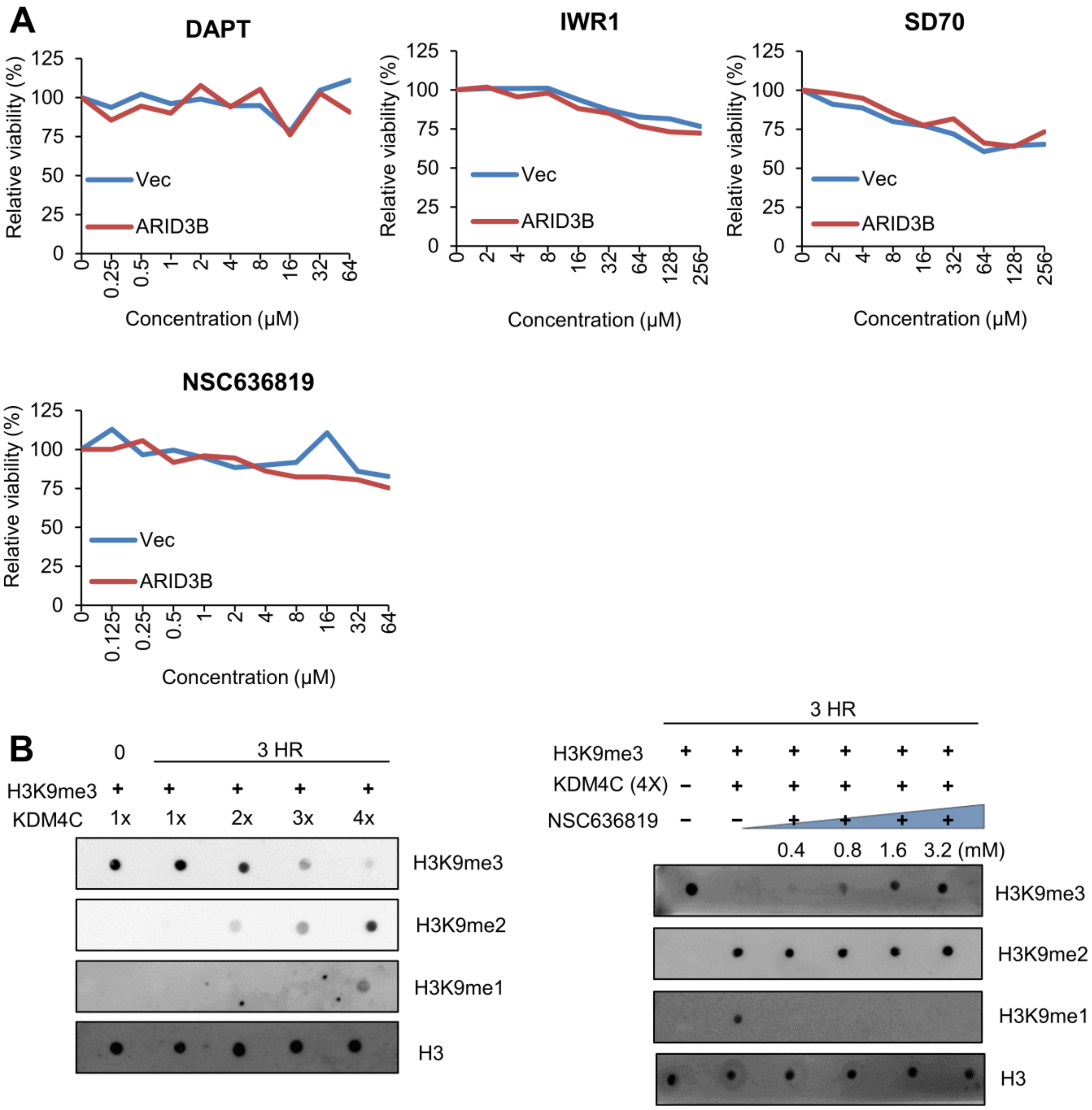

Figure S7. The effect of different inhibitors on the survival of HT-29 cells and the influence of the KDM4 inhibitors on H3K9 demethylation. **A** MTT assay for examining the survival of HT29-ARID3B vs. HT29-Vec cells treated with different inhibitors for 24 h. One representative experiment out of three independent experiments is shown. **B** In vitro demethylation assay. The H3K9me3 peptide was co-incubated with different amounts of purified KDM4C (left), or purified KDM4C with different concentrations of NSC636819 as indicated (right), at 37 °C for 3 h. The level of H3K9me3, H3K9me2, and H3K9me1 were visualized by dot blot analysis.

Supplementary Tables

Table S1. Primers for cloning of knockout constructions and plasmid information

**Name**	**Primer sequence**	**comments**
pcDNA-HA	N. A	The plasmid was kindly provided by Dr. Tien-Shun Yeh (J. Biol. Chem. 278: 41963–41969, 2003).
pcDNA-HA-N1IC		
pJH-23A		The plasmid was kindly provided by Dr. Tien-Shun Yeh (National Yang-Ming University), which was constructed by Dr. Diane Hayward (MOLECULAR AND CELLULAR BIOLOGY, 16: 952–959, 1996). The pJH-23A contains the 4x wt CBF1 Luc sites (GTGGGAA).
pJH-23A-mut		pJH-23A-mut contains 4x mutant CBF1 Luc sites (CTCGCCA)
Cas9-ARID3B(F)	caccgactctcctttccgccacagc	The sgRNA oligo was annealing and cloning into digested vector lentiCRISPR V2 (FastDigest BsmBI)
Cas9-ARID3B(R)	aaacgctgtggcggaaaggagagtc	
pLKO.1-control	TRCN0000231722	The shRNA plasmids were purchased from the National RNAi Core Facility of Taiwan for gene silencing.
PTGS2#33	TRCN0000045533	
PTGS2#37	TRCN0000045537	

Table S2. Primer list for quantitative PCR

Name	Sequence
GAPDH (F)	aaggtcggagtcaacggatttg
GAPDH (R)	ccatgggtggaatcatattggaa
CD44 (F)	ccagatggagaaagctctga
CD44 (R)	gtcatactgggaggtgttgg
MSI1(F)	cggtgaaactctggctagacag
MSI1(R)	gcaaaccgtagatgctcaggga
BMI1(F)	tccacaaagcacacacatca
BMI1(R)	ctttcattgtcttttccgcc
LRIG1(F)	caaaaccagcagggtcaatc
LRIG1(R)	acttgcgctggggactc
TERT(F)	atcagccagtgcaggaactt
TERT(R)	agctgacgtggaagatgagc
ASCL2(F)	cgcctactcgtcggacgacag
ASCL2 (R)	gccgctcgctcggcttccg
OLFM4 (F)	atctgcctcttcaggcgcat
OLFM4 (R)	ccccaggtttcttccaggca
SOX9 (F)	aggaagctcgcggaccagtac
SOX9 (R)	ggtggtccttcttgtgctgcac
Hes1 (F)	ggaaatgacagtgaagcacctcc
Hes1 (R)	gaagcgggtcacctcgttcatg
COX2 (F)	ggtgggaacagcaaggatt
COX2 (R)	ccctcagacagcaaagccta
LRIG1 (F)	gtaggttcggcaagtcctca
LRIG1 (R)	gacctgccctcctggac
OLFM4 (F)	cacactaattaattggacatattccct
OLFM4 (R)	gtggacagagtggaacgctt
LGR5 (F)	gtttcccgcaagacgtaact
LGR5 (R)	cagcgtcttcacctcctacc
CD274(F)	tgccgactacaagcgaattactg
CD274 (R)	ctgcttgtccagatgacttcgg

Table S3. Primary antibody list

Antibody name	Cat No.	Clone number*	Species	Assay	Condition
ARID3B	Bethyl, A302-564A	NA	R	western,ChIP, IHC	1000x for western, 1800x for IHC,5ug for ChIP; 4℃ o/n,200x; 4℃ o/n
ARID3B	Abnova, H00010620-B01P	NA	M	EMSA	2ug for EMSA; 4℃ o/n
HES1	Cell signaling, 11988	NA	R	IHC	600x for IHC
HES1	Santa cruz, sc-25392	H-140	R	western	500x ; 4℃ o/n
PD-L1	Cell signaling, 13684	E1L3N	R	IHC	200x ; 4℃ o/n
Anti-RBP-JK	Millipore, MABE982	1F1	R	ChIP	5ug for ChIP; 4℃ o/n
COX-2	Cayman,160106	NA	R	western	200x; 4℃ o/n
NICD	Cell signaling, 4147	D3B8	R	western	1000x ; 4℃ o/n
β-catenin	BD-610153	NA	M	western	1000x ; 4℃ o/n
phoshpho-β-catenin	Cell signaling, 9562	NA	R	western	1000x ; 4℃ o/n
GLI2	Santa cruz, sc-20291	NA	G	western	200x; 4℃ o/n
α-tubulin	Sigma, T6199	NA	M	western	3000x; 4℃ o/n
JNK	Cell signaling, 9252	NA	R	western	1000x ; 4℃ o/n
P-JNK(Thr183/Tyr185)	Cell signaling, 9251	NA	R	western	1000x ; 4℃ o/n
AKT	Cell signaling, 9272	NA	R	western	1000x ; 4℃ o/n
P-AKT(Ser473)	Cell signaling, 9271	NA	R	western	1000x ; 4℃ o/n
ERK	Cell signaling, 4695	137F5	R	western	1000x ; 4℃ o/n
P-ERK	Cell signaling, 4370	D13.14.4E	R	western	1000x ; 4℃ o/n
P-STAT3 (Y727)	Cell signaling, 9136	6E4	M	western	1000x ; 4℃ o/n
P-STAT3 (Y705)	Cell signaling, 9145	D3A7	R	western	1000x ; 4℃ o/n
STAT3	Cell signaling, 9139	124H6	M	western	1000x ; 4℃ o/n
P-STAT1(Tyr701)	Cell signaling, 9167	58D6	R	western	1000x ; 4℃ o/n
STAT1	Cell signaling, 14994	D1K9Y	R	western	1000x ; 4℃ o/n
OCT4	abcam, ab19857	NA	R	western	1000x ; 4℃ o/n
Nanog	abcam, ab62734	NA	R	western	500x ; 4℃ o/n
Sox2	Cell signaling, 2748	NA	R	western	1000x ; 4℃ o/n

H3K9me1	Biovison,6804-50	NA	R	western	1000x ; 4°C o/n
H3K9me2	abcam, ab1220	NA	M	western	1000x ; 4°C o/n
H3K9me3	abcam, ab8898	NA	R	western,ChIP	1000x for western, 1800x for IHC,5ug for ChIP ; 4°C o/n
Histone 3	GeneTex, GTX122148	NA	R	western	2000x ; 4°C o/n
β-actin	Sigma, A5441	AC-15	M	western	2000x ; 4°C o/n
KDM4C	Novus, NBD-49600	NA	R	western,ChIP	1000x for western, 2400x for IHC,5ug for ChIP; 4°C o/n
HA.tag	Upstate, 05-904	NA	M	western,IP	1000x for western,5ug for IP; 4°C o/n
Myc-tag	Thermo, MA1-21316	Myc.A7	M	western	1000x ; 4°C o/n
mouse IgG	Santa cruz, sc-2025	NA	M	IP, ChIP	2ug for IP ; 4°C o/n
rabbit IgG	Santa cruz, sc-2027	NA	R	IP, ChIP	2ug for IP ; 4°C o/n
CD44-PE	Biolegend, 103007	IM7	M	FC	200x; 4°C o/n

FC,Flow Cytometry; IHC, immunohistochemistry assay; IP, immunoprecipitration; EMSA,electrophoretic mobility shift assay ;ChIP, chromatin immunoprecipitration; o/n, overnight incubation, M, mouse; R, rabbit; G, goat; NA, not accessed.

Table S4. ChIP primer list

Primer mane	Sequence	Product length (bp)
HES1-chip (F)	gcgtgtctcctcctcccatt	108
HES1-chip (R)	cctggcggcctctatatata	
HES1-control-chip (F)	ccacacaggaaaaccctacg	164
HES1-control-chip (R)	tgccctgtcatgttctgaag	
COX-2-chip (F)	agggatcagacaggagagtg	106
COX-2-chip (R)	gtgggggcagggttttttac	
COX-2-control-chip (F)	ctattttctctcccttctcagc	107
COX-2-control-chip (R)	gaaaggagaattggactggttg	
LGR5-chip (F)	aatcttccaggcggaggctc	112
LGR5-chip (R)	acttgtcccctccctcctttc	
SOX9-chip (F)	caatcagctgcctgccaac	118
SOX9-chip (R)	ctccgctttcggctctcc	
CD274-chip (F)	agaatatcagggaccctgagcattc	107
CD274-chip (R)	cttcctcaaagttcctcgacataatg	
MSI1-chip (F)	aggcactgagtgggtccc	138
MSI1-chip (R)	gagaagccgccctcagag	

Table S5. Clinical characteristics of colorectal cancer patients for generating patient-derived xenografts.

**Patients characteristics**			
PDX case No.	case1	case21	case22
Gender:	Female	Female	Female
Birth date: (year)	1957	1938	1954
Date of diagnosis	2018/1/15	2017/11/23	2017/12/2
Date of last follow up:	2019/5/13	2019/5/14	2019/3/26
Treatment:Date of definite operation	2018/1/16	2017/12/4	2017/12/5
**Tumour characteristics**			
ASA SCORE：	1	3	3
Tumor location	Rectosigmoid junction	Sigmoid colon	Descending colon
TUMOR MARKERS: CEA:(initial)	11	4.6	10.3
CA-199:(initial)	138	7.69	34.03
Surgical resection: 1. Curative, 2. Palliative	1	2	1
Types of Operation	laparoscpic anerior resection	laparoscpic anerior resection	subtotal colectomy with lateral segmentectomy of liver
ADJUVANT THERAPY	Postoperative chemotherapy	Postoperative chemotherapy	Postoperative chemotherapy
Regimen_1: 5-FU, 2: Oxaliplatin, 3: Irinotecan, 4: Xeloda, 5: UFUR, 6: Cetuximab (Erbitux), 7: Bevacizumab (Avastin), 8: Aflibercept (Eylea), 9. Others:______	1,3,7	1,3,7	1,3,7
Histology	Adenocarcinoma	Adenocarcinoma	Mucinous adeno ca
Grade of differentiation: 1. Well 2. Moderately 3. Poorly .4. Undifferentiated 5.no data	2	2	2
Other pathological parameters. 1.Vascular invasion. 2.Lymphatic invasion. 3.Perineural invasion. 4.Isolated cancer nodule at mesentery.5. Inflammatory change around cancer (round cell infiltration) 6. Infiltrative invasive pattern of cancer tissue, 7. Signet ring cell component	1,2,4,6	4,5,6	1,2,4,6
Mucinous component______%	0	0	>75

No. of metastatic nodes/No. of total sampling nodes. /	11,26	1,15	5,50
Staging classification: (0:0 or Tis, 1:I, 2:IIA, 3:IIB, 4:IIC, 5:IIIA, 6: IIIB, 7:IIIC, 8:IVA, 9:IVB, according to AJCC7)	7	9	8
T	p4a	p4a	p3
N	2b	1a	2a
M	0	1	1
Size of tumor (according to the pathology report) __x___x__cm	3.5*3.2*X	4*3.2*X	12*5*X
MSI status	MSS	MSS	MSS
K-ras: (0: wild type)	1	0	1
K-ras: (1: mutant, codon change__________)	12		12
K-ras: (1: mutant, amino acid change __________)	c.35 g>a, Gly12Asp		c.35 g>t, Gly12Val
B-raf V600E: (0: wild type, 1: mutant)	0	0	0
N-ras: (0: wild type,)	0	1	0
N-ras: (1: mutant, codon change_________)	X	codon13, c.37 g>c, Gly13Arg	X
Condition of the patient at last follows up	Alive with colorectal cancer	Alive with colorectal cancer	Died of colorectal cancer

Table S6: cDNA microarray analysis of gene expression in HCT15-sg3B and HCT15-Ctrl

Data link as: https://drive.google.com/open?id=12ohKXFF4b9t3QrgA4WOl2lIP-3uR-JBB

Table S7: Functional analysis of gene expression in HCT15-sg3B and HCT15-Ctrl by Ingenuity Pathway Analysis

Data link as: https://drive.google.com/open?id=1dlaYMfQTel9KrW31AuEw-fbwIxEx4zfU

Table S8. The characteristics of 130 colorectal cancer patients

	n (%)
Gender	
Male	84(64.6)
Female	46(35.4)
Age (mean ± s.d.)	65.1±13.5
Location	
Proximal	58(44.6)
Distal	72(55.4)
CEA	
≧ 5	65(50.0)
< 5	50(38.5)
n.a.	15(11.5)
Perforation	
Presence	3 (2.3)
No	127(97.7)
Obstruction	
Presence	19(14.6)
No	111(85.4)
Stage	
I	14(10.8)
II	43(33.1)
III	40(30.8)
IV	33(25.4)
Mucin component	
≧50%	17 (13.1)
< 50%	110 (84.6)
n.a.	3 (2.3)
Grade of differentiation	
Well to moderate	111(85.4)
Poor	16(12.3)
n.a.	3(2.3)
Lymphovascular invasion	

Presence	30(23.1)
No	98(75.4)
n.a.	2(1.5)
Lymphocyte infiltration Presence	
Presence	20(15.4)
No	106(81.5)
n.a.	4(3.1)
Invasion pattern	
Infiltration	88(67.7)
Expansive	37(28.5)
n.a.	5(3.8)
ARID3B	
High	43(33.1)
Low	87(66.9)
KDM4C	
High	36(27.7)
Low	94(72.3)
HES1	
High	73(56.2)
Low	57(43.8)

Table S9. The demographics of 15 colorectal cancer patients with primary/metastatic tumor pairs

	n (%)
Gender	
Male	11(73.3)
Female	4(26.7)
Age (mean ± s.d.)	64.3±14.8
Location	
Proximal	2(13.3)
Distal	13(86.7)
CEA	
≧ 5	8 (53.3)
< 5	3 (20.0)
n.a.	4 (26.7)
Grade of differentiation	
Well to moderate	13 (86.7)
Poor	2 (13.3)
Lymphovascular invasion	
Presence	6(40.0)
No	9(60.0)
Lymphocyte infiltration	
yes	3(20.0)
no	12(80.0)
Invasion pattern	
Infiltration	14(93.3)
Expansile	1(6.7)
ARID3B (primary tumor)	
High	4 (26.7)
Low	11 (73.3)
PD-L1 (primary tumor)	
High	7 (46.7)
Low	8 (53.3)
PD-L1 intensity (primary tumor)	
0	6 (40.0)

1	4 (26.7)
2	4 (26.7)
3	1 (6.7)
ARID3B (metastatic liver tumor)	
High	13 (86.7)
Low	2(13.3)
PD-L1 (metastatic liver tumor)	
High	13 (86.7)
Low	2 (13.3)
PD-L1 intensity (metastatic liver tumor)	
0	0
1	9 (60.0)
2	5 (33.3)
3	1 (6.7)

*high PD-L1 is defined as having PD-L1 expression on > 50% of TC

PD-L1 expression score: 0 (absent), 1 (weak), 2 (moderate) or 3 (strong)

ARID3B: high: IRS 4~12; low: IRS 0~3

Table S10. The correlation between expressions of ARID3B, KDM4C, and HES1 in CRC patients

	KDM4C			HES1		
	Low (%)	High (%)	p	Low (%)	High (%)	p
ARID3B						
Low	69(73.4)	18(50.0)	0.011	44(77.2)	43(58.9)	0.028
High	25(26.6)	18(50.0)		13(22.8)	30(41.1)	
KDM4C						
Low				48(84.5)	46(63.0)	0.007
High				9(15.5)	27(37.0)	

Table S11.The correlation of expressions of ARID3A vs ARID3B, KDM4C, HES1, and CD44 in CRC patients

	ARID3A		
	Low	High	p
ARID3B			
Low (%)	32(37.2)	54(62.8)	0.111
High (%)	10(23.3)	33(76.7)	
KDM4C			
Low (%)	36(38.7)	57(61.3)	0.017
High (%)	6(16.7)	30(83.3)	
HES1			
Low (%)	21(37.5)	35(62.5)	0.294
High (%)	21(28.8)	52(71.2)	
CD44			
Low (%)	22(37.3)	37(62.7)	0.889
High (%)	17(38.6)	27(61.4)	

ARID3A, KDM4C, HES1, CD44: high: 2 = positive, mild expression (4-8); 3 = positive, strong expression(9-12); low: 0 = negative (0-1); 1 = positive, weak expression (2-3)

ARID3B: high: 3 = positive, strong expression (9-12); low: 0 = negative (0-1); 1 = positive, weak expression (2-3), 2 = positive, mild expression (4-8)

Table S12.The correlation of ARID3B vs PD-L1 in 15 pairs of matched primary-liver metastatic CRC samples

	PD-L1 proportion*			PD-L1 expression intensity				
	High	Low	p	0	1	2	3	p
ARID3B								
High (%)	17	0	0.001	0	8(47.1)	7(41.2)	2(11.8)	0.011
Low (%)	3(23.1)	10(76.9)		6(46.2)	5(38.5)	2(15.4)	0	

*high PD-L1 is defined as having PD-L1 expression on ≥50% of TC

PD-L1 expression score: 0 (absent), 1 (weak), 2 (moderate) or 3 (strong).

ARID3B: high: IRS 4~12; low: IRS 0~3
